# Divergent kinase regulates membrane ultrastructure of the *Toxoplasma* parasitophorous vacuole

**DOI:** 10.1073/pnas.1816161116

**Published:** 2019-03-08

**Authors:** Tsebaot Beraki, Xiaoyu Hu, Malgorzata Broncel, Joanna C. Young, William J. O’Shaughnessy, Dominika Borek, Moritz Treeck, Michael L. Reese

**Affiliations:** ^a^Department of Pharmacology, University of Texas, Southwestern Medical Center, Dallas, TX 75390;; ^b^Signalling in Apicomplexan Parasites Laboratory, The Francis Crick Institute, NW1 1AT London United Kingdom;; ^c^Department of Biophysics, University of Texas Southwestern Medical Center, Dallas, TX 75390;; ^d^Department of Biochemistry, University of Texas Southwestern Medical Center, Dallas, TX 75390

**Keywords:** kinase, pseudokinase, phosphorylation, host–pathogen interaction, chaperone

## Abstract

Apicomplexan parasites replicate within a protective organelle, called the parasitophorous vacuole (PV). The *Toxoplasma gondii* PV is filled with a network of tubulated membranes, which are thought to facilitate trafficking of effectors and nutrients. Despite being critical to parasite virulence, there is scant mechanistic understanding of the network’s functions. Here, we identify the parasite-secreted kinase WNG1 (With-No-Gly-loop) as a critical regulator of tubular membrane biogenesis. WNG1 family members adopt an atypical protein kinase fold lacking the glycine rich ATP-binding loop that is required for catalysis in canonical kinases. Unexpectedly, we find that WNG1 is an active protein kinase that localizes to the PV lumen and phosphorylates PV-resident proteins, several of which are essential for the formation of a functional intravacuolar network. Moreover, we show that WNG1-dependent phosphorylation of these proteins is required for their membrane association, and thus their ability to tubulate membranes. Consequently, WNG1 knockout parasites have an aberrant PV membrane ultrastructure. Collectively, our results describe a unique family of *Toxoplasma* kinases and implicate phosphorylation of secreted proteins as a mechanism of regulating PV development during parasite infection.

## Significance

Proteins are chemically modified after translation to regulate their functions. Protein kinases are enzymes that modify proteins with phosphate molecules. Using bioinformatics, we identified an unusual family of kinases (WNG kinases) that lack a structural motif, called the Gly-loop, which is absolutely required for the activity of all previously described kinases. Nevertheless, we found that the most conserved WNG kinase, WNG1, is catalytically active. The WNG kinases are only found in certain intracellular parasites, such as the human pathogen *Toxoplasma gondii*. We show that the founding member of the family, WNG1, phosphorylates parasite proteins that regulate membrane structure, and is therefore required for the proper biogenesis of the *Toxoplasma* parasitophorous vacuole, a structure essential for the parasite to cause disease.

Protein phosphorylation is the most common posttranslational modification in eukaryotic cells. The addition and removal of specific phosphates is a key mediator of cellular information processing and signal transduction. Phosphorylation is catalyzed by protein kinases, which form one of the largest families of enzymes in mammals (1). The interface between an intracellular pathogen and its host cell is a special case in cellular signaling that defines both a pathogen’s ability to manipulate its host and the host’s ability to respond to and control the pathogen. The parasite *Toxoplasma gondii* is one of the most successful pathogens in the world, as it can infect virtually any cell type of almost all warm-blooded animals, including approximately one-third of humans worldwide (2). *Toxoplasma* directly manipulates signaling at the host–pathogen interface by secreting a variety of effector proteins (3, 4), including ∼50 protein kinases and pseudokinases (5, 6). However, the functions of most of these effectors are unknown.

One vital role for these secreted kinases is to maintain the parasite’s replicative niche within its host cell. Like many intracellular pathogens, *Toxoplasma* survives in a specialized membranous organelle, called the parasitophorous vacuole (PV). This vacuole is maintained as distinct from host endosomal trafficking and is protected from fusion with host lysosomes (7). Disruption of the PV membrane by host immune defenses leads to parasite death (8, 9), and the parasite has evolved effector molecules that can protect it from such host attacks (10, 11). Far from being an impermeable wall, however, the parasite selectively exports (12) and imports (13, 14) molecules across the PV membrane.

One of the most striking features of the PV is the intravacuolar network (IVN) of membranous tubules of 20- to 50-nm diameter that appear to bud from the PV membrane into the vacuolar lumen (15). Notably, the inside of the tubules is topologically contiguous with the host cytosol (15, 16). The IVN has been associated with diverse phenomena, including nutrient uptake via trafficking of host-derived vesicles (17, 18), “ingestion” of soluble host proteins by the parasite (19), protection from antigen presentation (20), and a means by which parasite effectors localize to the PV membrane (21) and thus protect its destruction by host immune effectors (22). The dense granule proteins GRA2 and GRA6 are required for IVN biogenesis and parasites that lack either protein grow in vacuoles without the well-structured membranous tubules. While IVN-deficient parasites grow normally in in vitro cell culture (23, 24), they have strongly attenuated virulence in a mouse model of infection (25).

The PV is thus a complex cellular compartment that mediates sophisticated, multidirectional trafficking, although the molecules that regulate its functions are largely a mystery. Many of the known components of the PV, and of the IVN in particular, are highly phosphorylated after they have been secreted from the parasite (26). About one-third of the *Toxoplasma* kinome contains signal peptides but lack transmembrane domains, and are thus predicted to be secreted. Most of these kinases belong to a parasite-specific family that includes a number of virulence effectors (10, 27, 28) secreted into the host cytosol from the parasite rhoptries during invasion (29), and have been dubbed the “rhoptry kinase” (ROPK) family. A previous bioinformatic effort annotated the majority of predicted secreted kinases in *Toxoplasma* as ROPKs (5). Notably, vertebrate or ROPK effector kinases localized in the host cytosol cannot access PV-resident proteins on the luminal side of the PV membrane. However, two members of the ROPK family, ROP21/27, were recently found to be secreted into the PV lumen, rather than localizing to the rhoptries (30). Because ROP21/27 are expressed mainly during the chronic stage of the parasite (30), they are unlikely to function in the regulation of processes during the acute stage, such as the biogenesis of the IVN.

In the present work, we identify a specialized family of kinases that lack the glycine-rich loop that is critical for nucleotide-binding in canonical kinases, leading us to name them the With-No-Gly-loop, or WNG, family. These WNG kinases are conserved throughout the coccidian family of parasites to which *Toxoplasma* belongs and are secreted into the PV. We solved the crystal structure of a family member which demonstrates that the N-lobe of the kinase does indeed lack the structural elements that form the Gly-loop. We found that at least one member of the family, WNG1/ROP35, is catalytically active, and we identified a number of proteins associated with the IVN membrane as phosphorylated in a WNG1-dependent manner. Finally, we demonstrated that loss of these phosphorylation sites correlates with aberrant PV ultrastructure, likely due to the loss of membrane association of proteins that drive the biogenesis of IVN tubules. Taken together, our data show the WNG family of kinases mediates specialized functions in regulating the proteins that create and maintain the IVN tubules, which are crucial to the coccidian host–parasite vacuolar interface.

## Results

### Identification of a Divergent Family of Coccidian-Secreted Kinases That Lack the Canonical Glycine-Rich Loop.

**Fig. 1.**
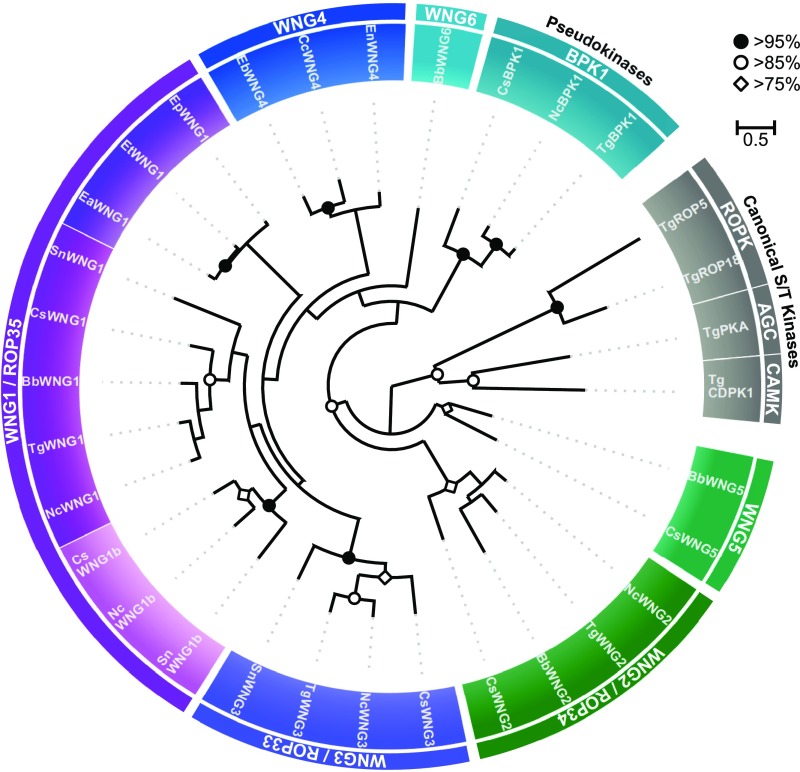
The WNG kinases comprise a phylogenetic clade that is distinct from canonical protein kinases. A maximum-likelihood phylogenetic tree was estimated from the multiple sequence alignment of the indicated kinases. Bootstrap values are indicated as black circles (>95%), white circles (>85%), and white diamonds (>75%). Species: *Bb*, *Besnoitia besnoiti*; *Cc*, *Cyclospora cayetanensis*; *Cs*, *Cystoisospora suis*; *Ea/Eb/Ep/Et*, *Eimeria* spp.; *Nc*, *Neospora caninum*; *Sn*, *Sarcocystis neurona*; *Tg*, *Toxoplasma gondii*.

We reasoned that regulatory phosphorylation of PV-resident proteins would most likely be carried out by a conserved resident protein kinase that is secreted from the parasite’s dense granules. To identify potential PV-resident kinases, we compared the sequences of the predicted secreted kinases in *Toxoplasma*. We were surprised to find that a small family of parasite kinases appear to completely lack the glycine-rich, or P-loop, that is found in all canonical kinases and is required for binding the ATP in the active site (31, 32) (*SI Appendix*, Fig. S1). These kinases include three proteins annotated as ROPKs (ROP33, ROP34, and ROP35), and a pseudokinase, BPK1, that has previously been identified as PV resident and a component of the bradyzoite cyst wall (33). Phylogenetic analysis gave clear support for these proteins forming a clade that is distinct from canonical protein kinases (Fig. 1), including the parasite ROPKs. Furthermore, we identified members of this family in every species of coccidian parasite for which genomic sequence is available (Fig. 1 and *SI Appendix*, Table S1*C*), suggesting that they play an important role in the parasites’ pathogenic lifestyle. Notably, with the exception of the PV-resident kinases ROP21/27, the majority of ROPKs are not conserved throughout coccidian parasites (34). Given the lack of the glycine-rich loop and phylogenetic evidence that indicates that these proteins form a distinct clade, we propose that the family be named the WNG (With-No-Gly-loop) kinases.

### WNG Kinases Are Secreted into the Parasitophorous Vacuole.

**Fig. 2.**
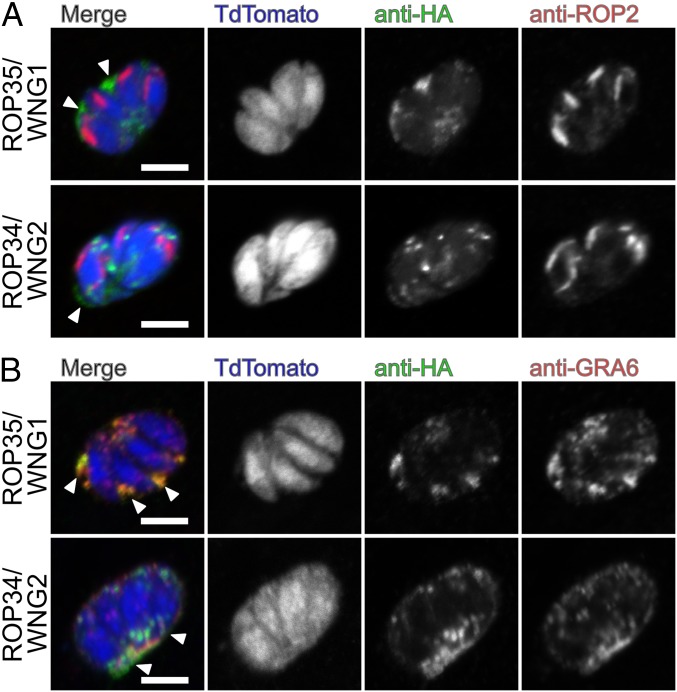
WNG kinases are secreted into the PV lumen: 0.5-μm confocal slices of ROP35/WNG1-3xHA or ROP34/WNG2-3xHA infected cells transiently transfected with TdTomato (blue) and stained with anti-HA (green) and either (*A*) the rhoptry marker anti-ROP2 (red) or (*B*) the dense granule/IVN marker anti-GRA6 (red). Arrowheads indicate secreted, PV-localized signal. (Scale bars, 5 μm.)

As noted above, BPK1 has previously been identified as a PV-resident pseudokinase (33). We thus sought to assess the localization of other WNG kinases and concentrated on the most divergent members of the family in *Toxoplasma*: ROP34 and ROP35 (Fig. 1). We engineered parasite strains in which the endogenous copies of each of ROP34 and ROP35 were expressed in frame with a 3xHA tag. While both proteins appeared to be secreted into the PV, neither ROP35 nor ROP34 colocalized with the rhoptry marker ROP2 (Fig. 2*A*), although ROP35 colocalized well with the dense granule marker GRA2 after secretion into the PV (Fig. 2*B* and *SI Appendix*, Fig. S2). However, both ROP34 and ROP35 appeared to colocalize with puncta of the dense granule marker GRA5 in extracellular parasites (*SI Appendix*, Fig. S2), suggesting that they are dense granule proteins. While these data appear inconsistent with the reported localization of ROP35 to the parasite PV via rhoptry secretion (35), we note that the previous report did not colocalize ROP35 with a known rhoptry marker, nor did it analyze endogenously tagged protein, both of which could lead to misinterpretation of the protein’s endogenous localization. Furthermore, both ROP34 and ROP35 have recently been identified as proteolytically processed by the Golgi-resident ASP5 protease (36), which appears to act exclusively on dense granule proteins. Because the “ROP” designation was originally created to indicate localization rather than function (37), we propose that the WNG kinases be renamed to avoid confusion with the unrelated ROPK family. Given its high conservation (Fig. 1 and *SI Appendix*, Table S1*C*), we propose ROP35 be renamed WNG1, and other family members annotated as in Fig. 1 and *SI Appendix*, Table S1*C*.

### The Crystal Structure of TgBPK1 Reveals a Noncanonical Active Site That Lacks the Gly-Loop.

**Fig. 3.**
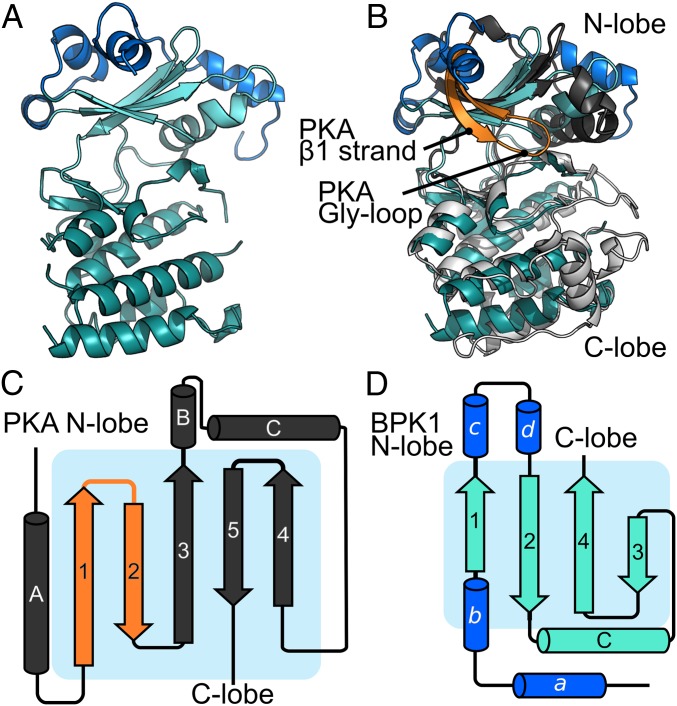
The structure of TgBPK1 reveals an atypical kinase fold lacking the Gly-loop. (*A*) Stereoview of the TgBPK1 structure. The N-lobe is in cyan, C-lobe colored in teal, and the helical “lid” that is unique to the WNG family is colored blue. (*B*) Superposition of the TgBPK1 structure with that of PKA (1ATP). TgBPK1 is colored as in *A*. The N-lobe of PKA is dark gray, C-lobe is light gray, and β-strands that sandwich the Gly-loop are orange. Cartoon highlighting the differences between the N-lobes of (*C*) PKA and (*D*) TgBPK1, colored as in *B*. Note the difference in the order of the N-lobe β-strands in PKA vs. TgBPK1.

While the Gly-loop is thought to be both a critical catalytic and structural element of the protein kinase fold, a number of unusual kinases have been demonstrated to have either adapted a canonical kinase fold to perform a specialized noncatalytic function (11, 38, 39), or to use an atypical fold and active site to catalyze phosphoryl transfer (40, 41). We therefore sought structural information to better understand the topology of the WNG kinase fold. While we were unable to crystallize an active WNG kinase, we readily obtained crystals of the *Toxoplasma* pseudokinase BPK1 (bradyzoite pseudokinase 1). We solved the structure of BPK1 to 2.5-Å resolution (Fig. 3*A* and *SI Appendix*, Table S2). Like WNG1, BPK1 is secreted into the lumen of the PV (33) and is a clear member of the WNG family (Fig. 1). As such, BPK1 shares both primary identity and predicted secondary structure with other WNG kinases throughout its sequence (*SI Appendix*, Fig. S1*A*), indicating that its structure would provide faithful insight into the WNG kinase fold.

The BPK1 structure revealed a divergent kinase fold in which the Gly-loop and the first β-strand that stabilizes it (β1 in PKA nomenclature) have been replaced by a helical extension that packs against the top of the N-lobe of the kinase (Fig. 3). Remarkably, not only do the WNG kinases lack a Gly-rich primary sequence, the structural elements that compose the motif have been replaced, resulting in a reorganized N-lobe architecture (Fig. 3 *C* and *D*). The core of the kinase fold, however, is remarkably well conserved, supporting our phylogenetic data (Fig. 1), which suggest the WNG family diverged from a canonical Ser/Thr kinase fold. Two salt bridges help stabilize the BPK1 N-lobe within the pseudoactive site, including the bridge between the conserved αC-helix Glu and VAIK-Lys (*SI Appendix*, Fig. S3*A*). Notably, the lack of the Gly-loop and β1-strand creates an active site that is much more open than that of a canonical kinase, such as PKA (*SI Appendix*, Fig. S3 *B* and *C*).

**Fig. 4.**
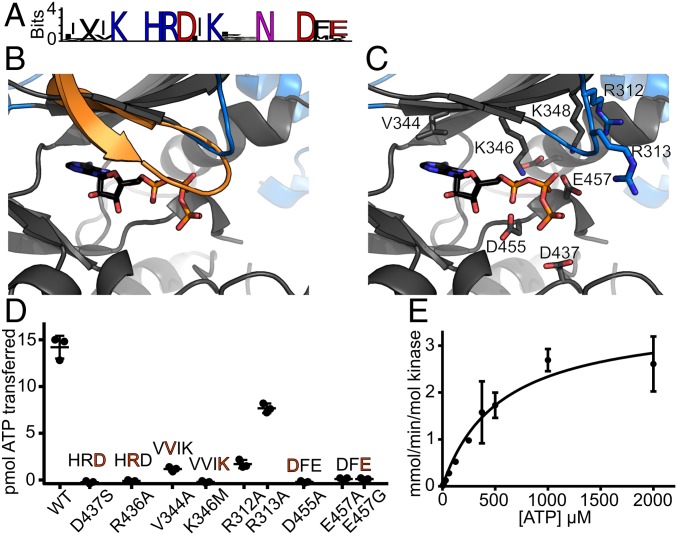
WNG1 has adapted its active site to catalyze phosphoryl transfer without a Gly-loop. (*A*) Sequence logos of the WNG kinase VAIK, HRD, and DFG motifs indicate conservation of critical catalytic residues. (*B*) A homology model of the WNG1 structure based on the BPK1 crystal structure, (gray and blue) has been superimposed with the structure of PKA (1.96-Å backbone rmsd; 529 atoms compared). For clarity, only the PKA Gly-loop (orange) and bound nucleotide are shown. (*C*) A model of the WNG1 active site structure, colored as in *B*. Bound ATP has been modeled based on superposition of the PKA structure. Residues that comprise either canonical motifs or WNG-specific substitutions are annotated and shown as sticks. (*D*) Kinase activities of WT WNG1 and the indicated mutant proteins using MBP as a protein substrate, quantified by ^32^P scintillation. Motifs altered by the mutants are shown above the data points. (*E*) A representative Michaelis–Menten fit of in vitro kinase assays of WNG1 using MBP as a substrate while varying ATP concentration.

While BPK1 is a confirmed pseudokinase that cannot bind nucleotide (42), the other canonical motifs essential for catalysis have been conserved in other WNG family members (Fig. 4*A* and *SI Appendix*, Fig. S1*A*), suggesting they may be active kinases. To better understand how the WNG kinase active site has adapted to bind nucleotide and catalyze phosphoryl transfer without a Gly-loop, we modeled the WNG1/ROP35 active site using the structure of BPK1 as a template (Fig. 4 *B* and *C*). This model, together with analysis of sequence conservation among WNG1/ROP35 orthologs (*SI Appendix*, Fig. S4), confirmed that the core of the canonical active site appears largely conserved (Fig. 4*C*).

We expressed and purified the kinase domain of *Toxoplasma* WNG1/ROP35 and found that it robustly phosphorylated the generic substrate myelin basic protein (MBP) in an in vitro kinase assay. We verified that mutation of each of the canonical motifs that enable catalysis and Mg^2+^/ATP-binding (HRD*, VAIK*, D*FG) resulted in loss of kinase activity (Fig. 4*D*). We also identified three notable variations from typical motifs within the active site. First, we noted that while substitution of the Ala in the VAIK motif to a bulkier side chain usually interferes with ATP-binding, a Val appears to be preferred at this position in WNG family members. Mutation of V344A in WNG1/ROP35 reduced the specific activity of the kinase to ∼20% of WT (Fig. 4*D*), consistent with a requirement for repositioning the ATP within the WNG active site. Second, we noted a conserved stretch of basic residues in WNG1/ROP35 orthologs (R312/313 in *Toxoplasma*) that are placed near where the Gly-loop would lie (Fig. 4 *B* and *C* and *SI Appendix*, Fig. S4). We therefore reasoned that the side chains of these residues may form a degenerate Walker A motif-like cap (31), and help replace the Gly-loop function. Consistent with such a model, both R312A and R313A mutants exhibited reduced specific activity, although R313A showed a much less severe effect than R312A (Fig. 4*D*).

Finally, we noted that the WNG kinase Mg^2+^-coordinating DFG motif had an acidic residue (E457 in WNG1) replacing the Gly. As in our BPK1 structure (*SI Appendix*, Fig. S3*A*), the WNG1 E457 appears to form a salt-bridge with a conserved basic residue +2 from the VAIK Lys (Fig. 4*C*) (K348 in WNG1). This substitution is unusual for two reasons: (*i*) the DFG Gly is thought to be important for the regulation of many kinases, as it enables the peptide backbone to “flip” between two states, “DFG-in” and “DFG-out” (43, 44); (*ii*) the side chain of the Glu would be predicted to point toward the phosphates of the bound nucleotide (Fig. 4*C*), and would thus electrostatically clash. We reasoned that a clash may be prevented, however, if the residue was participating in Mg^2+^-coordination, as the Asp in the DFG does. The pseudokinase domain of metazoan RNaseL also has this unusual substitution, in this case, a DFD motif. The crystal structure of RNaseL pseudokinase demonstrated that both acidic residues in the DFD motif participate in Mg^2+^-coordination (45), helping to explain the protein’s unusually high affinity (1 μM) for ATP. We therefore tested whether mutation of WNG1/ROP35 E457 to either Gly or Ala would affect its activity, and found that both mutant proteins had severely attenuated activity that was not significantly different from the kinase-dead HRD D437S mutant (Fig. 4*D*).

We went on to determine that our recombinantly expressed WNG1 has an in vitro *K*_M,ATP_ of 520 ± 90 μM (Fig. 4*E*), using MBP as a substrate. Given the lack of the Gly-loop, which is a key ATP-binding element, it is unsurprising that this *K*_M,ATP_, is higher than the 10–100 μM reported for many canonical kinases (46). However, the mammalian kinases Src and Akt have reported *K*_M,ATP_ of ∼200 and 500 μM, respectively (46), indicating that our value for WNG1/ROP35 is consistent with an active kinase. Furthermore, the PV membrane is permeable to small molecules such as nucleotides (13, 14), and cellular ATP concentrations range between 2 and 5 mM (47), suggesting that PV nucleotide concentrations are well above that needed for activity with such an affinity for nucleotide.

Taken together, our structural and biochemical data suggest that WNG1/ROP35 and other family members are active protein kinases that have evolved multiple alterations to the active site to compensate for the lack of a Gly-loop. Furthermore, these broad structural changes imply an evolutionary pressure to reshape the protein structure to perform a specialized function.

### The IVN of Parasites Deficient in WNG1 Kinase Activity Is Unstable.

We next sought to identify potential functions for the WNG kinases. We chose to concentrate our efforts on WNG1 because it is conserved throughout coccidia (Fig. 1), concentrates within the PV lumen (Fig. 2), and is important for chronic infection in a mouse model of infection (48). We used double homologous recombination to knock out the WNG1 locus in the RH*Δku80Δhxgprt* background (*SI Appendix*, Fig. S5*A*). The resulting RH*Δwng1* parasites showed no obvious growth phenotype in normal culture conditions. We also generated WNG1-complemented strains by knocking a WT or kinase-dead (D437S; the HRD motif) copy of WNG1 into the empty Ku80 locus of the RH*Δwng1* strain. The kinase was expressed by its native promoter and in-frame with a C-terminal 3xHA. Both the active and kinase-dead complement strains expressed WNG1 at similar levels to the levels in the endogenously tagged parasite strain, and were appropriately localized to the vacuolar space (*SI Appendix*, Fig. S5*B*).

**Fig. 5.**
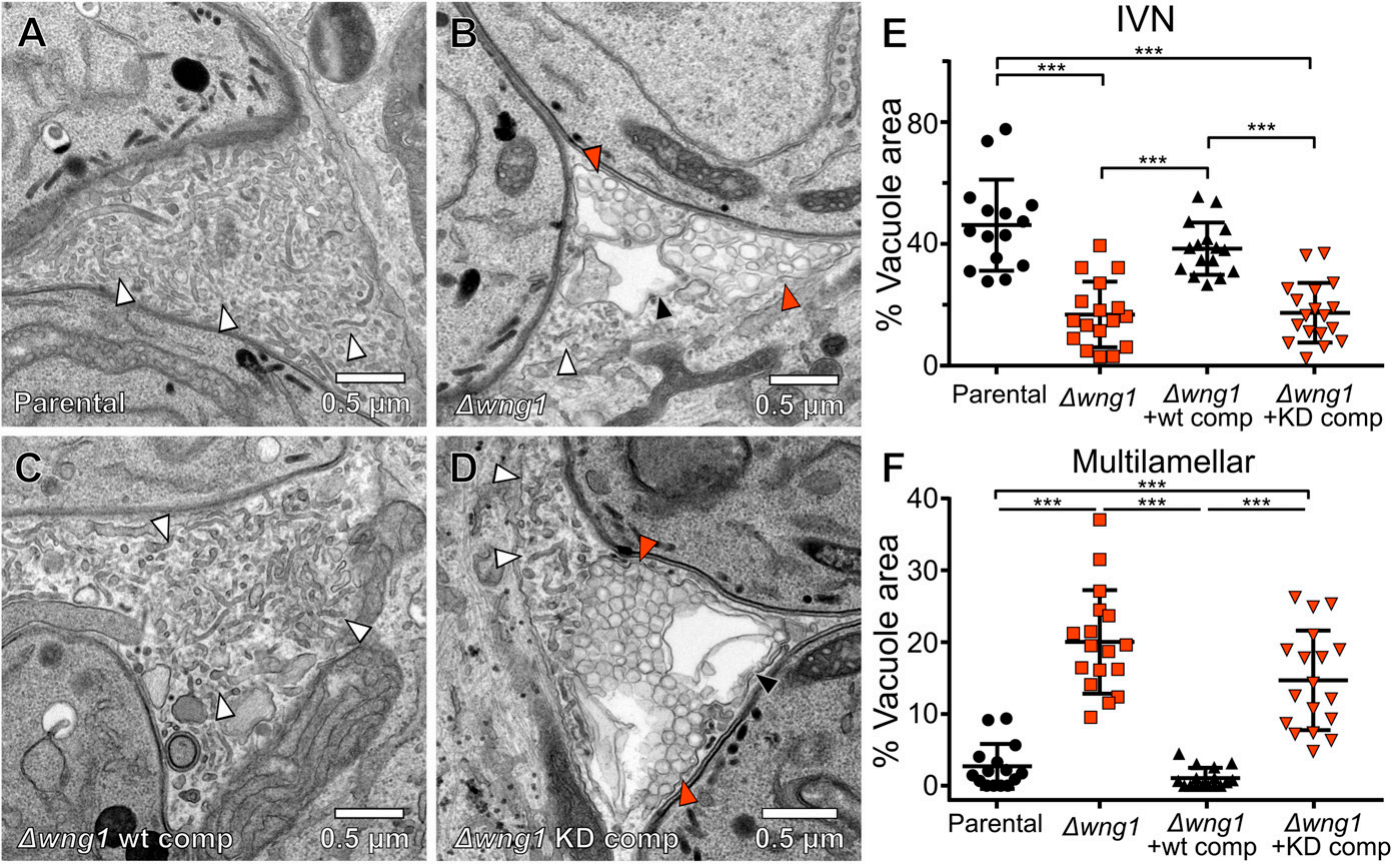
Vacuoles lacking active WNG1 kinase show disrupted IVN membranes. Representative transmission electron microscopic images of the (*A*) parental, (*B*) RH*Δwng1*, (*C*) RH*Δwng1* complemented with WT WNG1, and (*D*) kinase-dead complemented strains. IVN tubules are indicated with white arrowheads. Multilamellar vesicles are indicated with solid orange arrowheads. Multilamellar structures in which internal vesicles appear to have been lost during fixation are indicated with a black arrowhead in *B* and *D*. The relative area of each IVN tubules and multilamellar vacuole from EM images as in *A*–*D* were quantified in ImageJ. (*E* and *F*) Significance was calculated in Prism by ANOVA with Tukey’s test; ***P < 0.0001. Note that the parental vs. WT-comp and *Δwng1* vs. KD comp showed no significant difference.

To examine the ultrastructure within the vacuoles of parasites with and without active WNG1, we used transmission electron microscopy. We compared the vacuoles of human foreskin fibroblasts (HFFs) that had been infected for 24 h with either parental, RH*Δwng1*, or the complemented strains (Fig. 5 and *SI Appendix*, Fig. S5*C*). The IVN is a complex structure of branching membranous tubules that fills a large portion of the PV lumen (47). As expected, we observed a dense network of tubules filling the luminal space between the parental parasites (Fig. 5*A*). While we did observe regions with IVN tubules in RH*Δwng1* vacuoles, they have been largely replaced with unusual multilamellar structures containing many 70- to 150-nm diameter vesicles within a larger 0.5- to 2-μm membrane-delineated object (Fig. 5*B*). These multilamellar structures appear much less electron dense than the tubular network, suggesting a lower protein content. Consistent with this observation, the internal vesicles appear to have been lost in some structures (Fig. 5*B* and *SI Appendix*, Fig. S5*C*), potentially due to reduced cross-linking before plastic embedding. Importantly, we prepared samples from mutant parasite strains in parallel with a parental control. We never observed loss of tubular structures in the parental strains, suggesting that this phenotype is not an artifact of our preparation. While vacuoles of WT WNG1 complemented parasites were indistinguishable from the parental, those formed by the kinase-dead complemented strain exhibited the same loss of IVN tubules and its apparent replacement with large multilamellar vesicles (Fig. 5 *C* and *D*). These changes are quantified in Fig. 5 *E* and *F*. These data indicate that WNG1 phosphorylates one or more proteins involved in IVN biogenesis and stability, and that this phosphorylation is required for normal function.

### Quantitative Phosphoproteomics Reveals GRA Proteins as Candidate Substrates of WNG1.

**Fig. 6.**
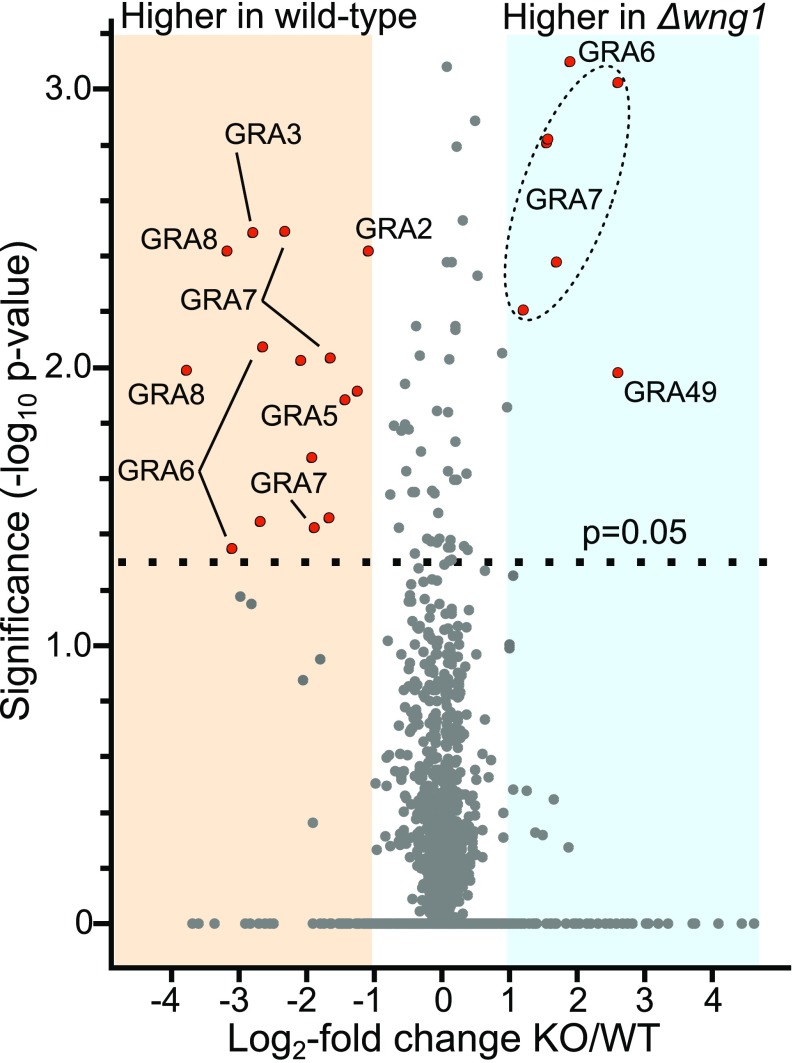
Overview of quantitative phosphoproteomics data. SILAC quantification of change in phosphosite abundance plotted against significance of change for 2,296 phosphosites in RH*Δwng1* versus parental parasites. See Dataset S1 for full dataset. Significantly changing phosphosites (*P* < 0.05 and −1 > log_2_ fold-change > 1) enriched in dense granule proteins are highlighted in red.

To identify potential substrates of WNG1, we compared the phosphoproteomes of the parental (WT) and RH*Δwng1* strains using stable isotope labeling with amino acids in cell culture (SILAC) quantitative mass spectrometry (MS) based proteomics as previously described (49). Briefly, we infected HFFs for 24 h with WT or RH*Δwng1* parasites previously grown in either “heavy” (H) or “light” (L) SILAC media. After cell lysis, we mixed the samples (H and L) in 1:1 ratio applying forward (*Δwng1*/WT), reverse (WT/*Δwng1*) as well as control labeling (WT/WT). This mixing strategy ensures that both systematic and technical errors due to stable isotope labeling can be identified, and results in high confidence of MS quantifications. Mixed lysates were then digested with LysC/trypsin and phosphopeptides enriched and fractionated as described in *Materials and Methods*. We prepared three biological replicates for WT vs. *Δwng1* samples and analyzed quantitative differences in the proteome and phosphoproteome between WT and mutant samples by MS. We identified 10,301 phosphosites for both human and *Toxoplasma* and obtained quantification (H/L ratios) for 8,755 of them. *Toxoplasma*-specific sites constituted 2,296 (∼30%) of all quantified sites (Dataset S1), which is a similar proportion of sites identified in previous studies using intracellular *Toxoplasma* parasites (26). To identify significantly changing sites between WT and RH*Δwng1* parasites a one-sample *t* test was performed applying the following parameters: *P* < 0.05 and |log_2_| fold-change > 1 (Fig. 6). Furthermore, phosphosite significance was also correlated with the SILAC control sample (WT/WT) and the proteome data to control for differential phosphorylation originating from the technical variation in the system and protein abundance, respectively (Dataset S1). We also identified a number of phosphorylation sites on proteins with consistent loss of phosphorylation in the RH*Δwng1* parasite strain that did not pass the *t* test significance test (Dataset S1). However, all phosphorylation sites close to the *P* value cut-off are predicted or known secreted proteins, indicating that the *P* value may be overly stringent in this case.

**Fig. 7.**
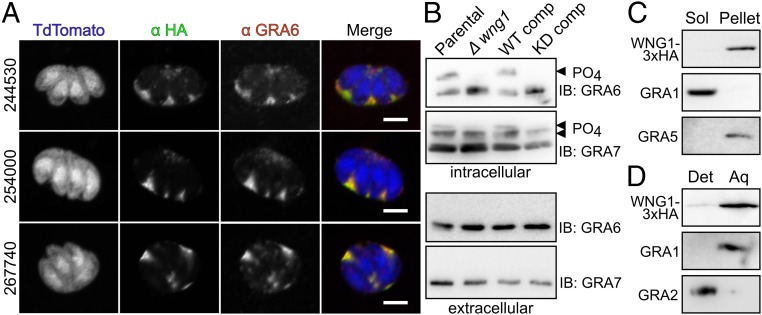
WNG1 and its substrates are membrane associated. (*A*) The 0.5-μm confocal slices of cells infected with parasites in which the indicated unannotated candidate substrates are endogenously 3xHA tagged (anti-HA; green), transiently transfected with TdTomato (blue), and costained with the dense granule and IVN marker GRA6 (red). (Scale bars, 5 μm.) (*B*) Western blot of lysates of extracellular parasites or cells infected (intracellular) with the indicated WT, knockout, or complement strains probed with anti-GRA6 and anti-GRA7 antisera. Phosphorylated bands are indicated with arrowheads. (*C*) Western blot of host and PV membranes that have been ultracentrifuged. WNG1-3xHA is detected with anti-HA, GRA1 and GRA2/GRA5 are used as soluble and membrane-associated controls, respectively. (*D*) Western blot of host and PV membranes that have been subjected to Triton X-114 partitioning between detergent (Det) and aqueous (Aq) phases.

We identified 10 proteins in which phosphorylation was significantly reduced between the parental and RH*Δwng1* samples (Fig. 6, Table 1, and Dataset S1). Among these candidate substrates were six proteins well-known to be associated with the PV membrane or IVN tubules (Table 1), including GRA2 and GRA6, which are essential for IVN biogenesis (47). Another hit, GRA37, was identified in a recent proteomics analysis of PV membrane proteins, and was found to colocalize with IVN markers (50). We identified three proteins with WNG1-dependent phosphorylation that have not been previously studied, and are therefore annotated as “hypothetical” in the genomic database (ToxoDB v32 gene models: TGGT1_244530, TGGT1_254000, and TGGT1_267740). We reasoned that if WNG1 is, indeed, a PV-resident kinase, WNG1-dependent phosphorylation should predict PV (and possibly IVN) localization. We therefore engineered strains in which the proteins were endogenously tagged at their C terminus with a 3xHA epitope. Immunofluorescence revealed that each of these proteins were secreted into the PV, and colocalized with dense granule markers both within the vacuolar lumen (Fig. 7*A*) and within the parasites (*SI Appendix*, Fig. S6*A*). We have thus annotated these three genes as encoding newly described dense granule proteins GRA47, GRA48, GRA49 (Tables 1 and 2).

**Table 1.** Phosphosites down-regulated in RH*Δwng1* vacuoles

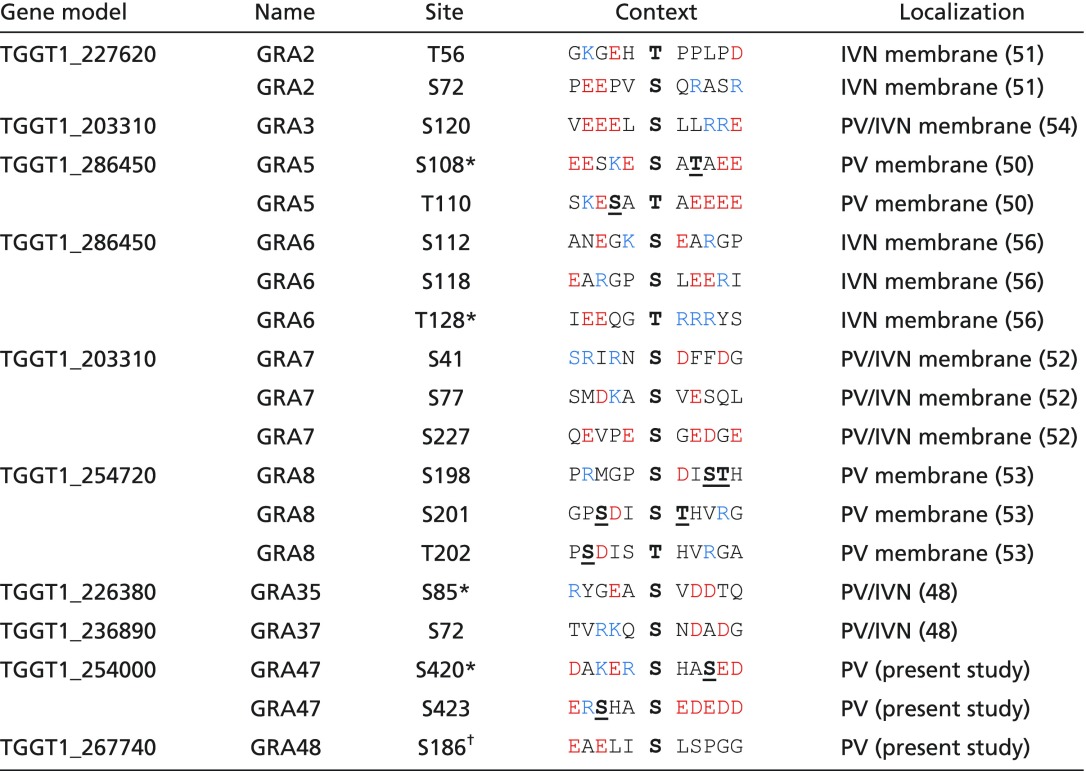

The sequence context of each of the phosphosites is indicated. Acidic residues are red, basic residues are blue. Note that some regions appear to be hyperphosphorylated in a WNG1-dependent manner. Such potential priming sites are indicated bolded, with bolded and underlined in the phosphosite context.

*Not significant in *t* test due to variability between replicates (*P* > 0.05) or quantified in only one type of labeling.

^†^Identified in preliminary dataset, not statistically significant in final data.

**Table 2.** Phosphosites up-regulated in WNG1-deficient vacuoles

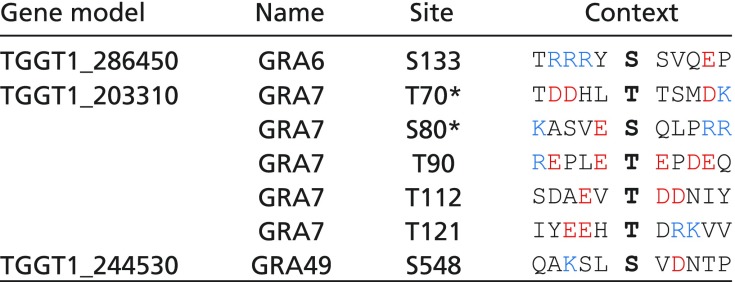

Table is formatted and colored as in Table 1.

*Not significant in *t* test due to variability between replicates (*P* > 0.05) or quantified in only one type of labeling.

In addition to the phosphosites that were down-regulated in the vacuoles lacking WNG1, we identified seven sites where phosphorylation was significantly increased in the RH*Δwng1* samples over parental (Table 2). These include one site on GRA6, five sites on GRA7, and one site on GRA49. The phosphorylated states of GRA6 and GRA7 in cells infected with WT parasites are readily distinguishable by SDS/PAGE and Western blot (17, 51) (Fig. 7*B* and *SI Appendix*, Fig. S6*B*). To confirm the changes in phosphorylation of these proteins, we blotted lysates of cells infected with either the parental or RH*Δwng1* strains (Fig. 7*B*). To demonstrate that these changes were due to the presence of WNG1, and to confirm the requirement of WNG1 kinase activity, we also assessed GRA6 and GRA7 phosphorylation in the WT and kinase-dead WNG1 complemented strains. The slower migrating, phosphorylated band of GRA6 was apparent in both parental and WT complemented lysates, but was undetectable in the knockout and kinase-dead complemented lysates (Fig. 7*B*). Consistent with our phosphoproteomics data, we observed a reduction in, but not complete loss of, phosphorylated GRA7 in the knockout and kinase-dead complemented parasites versus WT. These residual phosphorylated species are presumably the phospho-states listed in Table 2 that must be due to the activity of an unknown, additional kinase. In support of the idea that WNG1 is acting directly on these potential substrates, recombinant WNG1 robustly phosphorylated bacterially expressed GRA2, GRA6, and GRA7 in vitro (*SI Appendix*, Fig. S6*C*). Our data thus demonstrate that WNG1 is an active, PV-resident kinase required for the phosphorylation of luminal proteins associated with the PV and IVN membranes. Notably, each of the WNG1-dependent phosphosites we identified has been previously found exclusively in intracellular parasites (26) (Fig. 7*B*), indicating that phosphorylation is occurring only after secretion of the proteins into the PV.

Given that these candidate WNG1 substrates have been demonstrated to either associate with or integrate into PV membranes (51–56), we asked whether WNG1 itself was membrane associated once secreted into the PV. To test this, we mechanically disrupted an HFF monolayer that had been highly infected with WNG1-3xHA parasites. Intact parasites were separated from host and PV membranes by a low-speed (2,500 × *g*) spin, and the resulting supernatant was further separated by ultracentrifugation. WNG1, like the known integral membrane protein (and putative WNG1 substrate) GRA5, was found largely in the membrane-associated pellet (Fig. 7*C*). In parallel, we partitioned an aliquot of the same low-speed supernatant with Triton X-114 (57). In this assay, WNG1 partitioned in the aqueous phase (Fig. 7*D*)—indicating that it is a soluble protein that is membrane-associated—rather than integrating into the membrane directly. Such a nonintegral association of WNG1 with the PV membrane is consistent both with the lack of a predicted transmembrane, amphipathic helix, or another membrane association domain in the WNG1 sequence, and with our ability to purify soluble recombinant protein.

### Efficient Membrane Association of Proteins Involved in IVN Biogenesis Depends on WNG1 Kinase Activity.

**Fig. 8.**
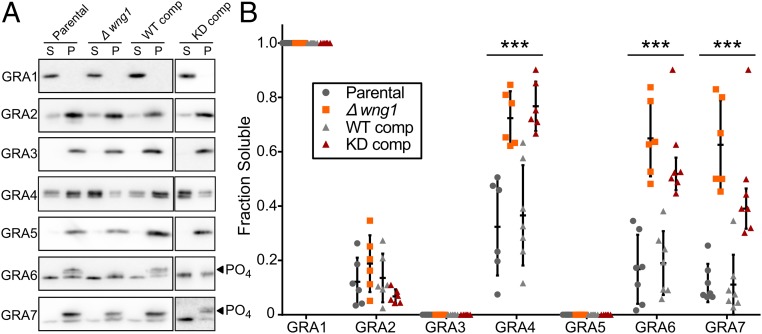
Membrane association of GRA proteins correlates with WNG1 kinase activity. (*A*) Representative Western blot of samples in which host and PV membranes were ultracentrifuged and the soluble (S) and pellet (P) fractions were separated by SDS/PAGE and probed with the indicated antisera. Phosphorylated bands are indicated with arrowheads. (*B*) Quantification of *n* = 6–7 biological replicates as in *A*. Significance was calculated by ANOVA with Tukey’s test in Prism; ***P < 0.001.

The trafficking of IVN-associated proteins is highly unusual. Many GRA proteins integrate amphipathic or transmembrane helices into the IVN membrane, but remain soluble while trafficking through the parasite secretory system (51, 52, 56), presumably by complexing with an unidentified chaperone. Notably, many of the WNG1-dependent phosphorylation sites are located in, or adjacent to, predicted helical regions of sequence (*SI Appendix*, Fig. S7*A*) that have been shown to be required for GRA membrane association (53, 58). We therefore reasoned that phosphorylation of substrates by WNG1 may help regulate the switch from soluble to membranous states of PV GRA proteins. To test this hypothesis, we assessed WNG1 membrane association by comparing fractionated lysates from parental RH*Δwng1*, and the kinase-active and kinase-dead complement strains. We prepared samples from six independent infections per condition, which were then separated by SDS/PAGE and analyzed by protein immunoblotting using antibodies recognizing various GRA proteins, as indicated in Fig. 8. We observed no difference in Triton X-114 partitioning for any of the strains (*SI Appendix*, Fig. S7*B*). We quantified the relative soluble amounts of each protein (Fig. 8*B*), which revealed a requirement for WNG1 kinase activity on IVN GRA membrane association. In particular, GRA4, GRA6, and GRA7 exhibited significant reductions in the fraction of protein that was PV membrane-associated in the RH*Δwng1* and kinase-dead samples. Notably, the phosphorylated forms of GRA6 and GRA7 are found exclusively in the membrane-associated fractions (Fig. 8*A*), and the loss of the slower migrating, phosphorylated states does not appear to result in a concomitant increase in the faster mobility (and presumably unphosphorylated) species at the membrane (*SI Appendix*, Fig. S7*C*). Taken together, our results suggest that WNG1-dependent phosphorylation of the GRA proteins promotes their association and is critical for the proper formation of the IVN.

## Discussion

We have identified an unusual family of parasite-specific protein kinases that divergently evolved from a canonical protein kinase fold and have lost the typical Gly-rich loop. We have demonstrated that, despite missing a structural element thought to be critical to nucleotide binding and catalytic activity, at least one of the WNG kinases can catalyze phosphoryl transfer. Through structural and biochemical analyses, we have delineated subtle changes to the kinase active site that facilitate its catalytic activity. We went on to show that the most conserved member of the family, WNG1/ROP35, is secreted by *Toxoplasma* into the PV, where it associates with the PV membranes. We found that WNG1 kinase activity is required for the phosphorylation of many of the proteins known to be associated with the PV and IVN membranes. Furthermore, loss of WNG1 kinase activity was correlated with a reduction in membrane association for a subset of the GRA proteins for which there are antibodies available. Finally, we found that parasite vacuoles deficient in catalytically active WNG1 have a substantial reduction in their IVN, suggesting that kinase activity is required for either the efficient formation or stability of the IVN membrane tubules.

The unusual WNG kinase fold raises the question: What may have been the evolutionary pressure that drove the divergence of the WNG family and loss of the Gly-loop? WNG1 is the most conserved member of the family, and appears to preferentially phosphorylate sites on proteins closely associated with the PV and IVN membranes. Moreover, many of the sites we identified are at or near predicted helices (*SI Appendix*, Fig. S7*A*) that have been previously implicated in GRA protein interaction with membranes (24, 52, 53, 58) or, in the case of GRA3, within a predicted coiled-coil. The rearrangement of the WNG active site has resulted in an unusually open active site (*SI Appendix*, Fig. S3 *B* and *C*) that may better accommodate such folded or otherwise sterically restricted substrates. The atypical “alpha” family of kinases (59) are also able to phosphorylate helical substrates, such as the coiled-coil domains of myosin heavy chains (60). The alpha kinases share no detectable sequence homology to canonical protein kinases despite their similar overall folds (61, 62). The active sites of alpha kinases differ in several ways from canonical protein kinases. As with the WNG kinases, the alpha kinases have a more open active site that would accommodate a helical substrate (62). In any event, a comprehensive understanding of the mechanisms of substrate recognition in atypical kinases, such as the WNG and alpha kinase families, will require structural studies of kinase:substrate complexes.

Notably, the phosphosites we identified as WNG1-dependent are not detectable in extracellular parasites (26) (Fig. 7*B*), indicating that WNG1 phosphorylates its substrates in the PV lumen rather than while trafficking through the parasite secretory system. Our phosphoproteomics data revealed both phosphosites that are lost in WNG1 knockout parasites, as well as a smaller number of up-regulated sites that were only detectable when WNG1 was missing. These data suggest that another kinase is capable of phosphorylating a subset of sites on the IVN GRA proteins, and its activity may be partially compensating for WNG1 loss. Alternatively, this other kinase activity may be acting in competition with that of WNG1. It is possible that these sites are phosphorylated by another member of the WNG family. However, we cannot rule out that WNG1 is acting indirectly on IVN GRA phosphorylation, for example, by activating another PV-resident kinase. Regardless, the data we present here are consistent with a role for WNG1-dependent phosphorylation in the regulation of protein–protein and protein–membrane interactions of PV-resident proteins. Unfortunately, very little is known about the biochemistry of the GRA proteins secreted into the PV, making it difficult to assess the relevance of individual phosphorylation sites. Nevertheless, elucidating the regulatory functions of WNG-dependent phosphorylation represents a rich avenue of future study.

**Fig. 9.**
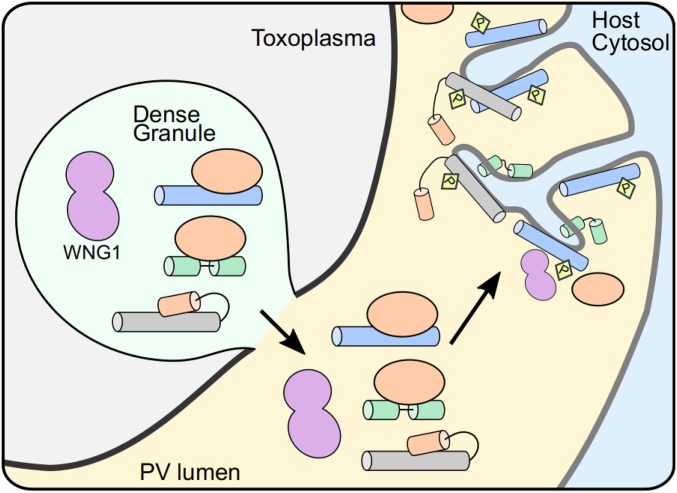
Model for WNG1 regulation of IVN GRA protein membrane association. Within the parasite secretory pathway, membrane-seeking GRA proteins (blue, green, and gray cylinders) are complexed with solubilizing proteins or domains (orange). Once secreted into the PV lumen, WNG1 is activated through an unknown mechanism and phosphorylates the GRAs, leading to their eventual insertion into the PV membrane and efficient stabilization of the IVN tubules.

The multilamellar vesicles we observe in vacuoles deficient in WNG1 kinase activity are reminiscent of structures that have been previously observed during the first steps of IVN biogenesis (15). While bacterially expressed GRA2 and GRA6, which we assume to be unphosphorylated, are sufficient to tubulate large unilamellar vesicles in vitro (20), it is possible that WNG1 kinase activity is required to ensure the efficiency of this process in cells. This may be explained by an apparent paradox that exists in GRA protein trafficking: GRA proteins that are destined to integrate into PV membranes traffic through the parasite secretory system as soluble entities (51–53), presumably in complex with an unknown solubilizing protein (Fig. 9). Such a switch ensures that the parasite’s intracellular and plasma membranes are protected from the tubulating activity of the GRA proteins. Removal of a solubilizing chaperone normally requires energy provided by ATP hydrolysis. There are no known chaperones secreted into the *Toxoplasma* PV. Consistent with a model in which WNG1 regulates membrane association of a subset of PV GRAs, we observed that each GRA4, GRA6, and GRA7 were substantially more soluble in the vacuoles of parasites deficient in WNG1 kinase activity. There is thus an intriguing possibility that the ATP used during WNG-mediated phosphorylation is providing the energy to dissociate a chaperoning interaction and drive membrane insertion of a subset of GRA proteins (Fig. 9). Such a noncanonical chaperoning mechanism is not without precedent. The mammalian neuropeptide 7B2 solubilizes the prohormone convertase 2 as it traffics to the Golgi (63), where 7B2 is phosphorylated by a resident kinase, resulting in release of the complex (64, 65).

Despite decades of study, the *Toxoplasma* PV remains a mysterious organelle. The major function identified for IVN-associated proteins is in IVN biogenesis, as the deletion of either GRA2 or GRA6 results in a complete loss of the structure (47). Such IVN-deficient parasites have been used to link the IVN to nutrient uptake (17–19) and immune evasion (20, 22), although the precise mechanisms and roles of the IVN have not been established in these processes. Consistent with the pleiotropic effects of disrupting the IVN, knockout of IVN-associated proteins strongly attenuates parasite virulence (25, 66). Infection of mice with WNG1 knockout parasites yields a substantially reduced cyst burden (48), which is consistent with the role we observed for WNG1 in IVN biogenesis and stability and the likely resulting pleiotropic effects on the parasite’s biology. Our discovery of potential regulatory phosphorylation may facilitate future work to associate specific GRA protein complexes with their biochemical functions and thus better delineate the roles of the IVN in parasite pathogenesis.

## Materials and Methods

### PCR and Plasmid Generation.

All PCR was conducted using Phusion polymerase (New England Biolabs) using primers listed in Dataset S2. Constructs were assembled using Gibson master mix (New England Biolabs). Point mutations were created by the Phusion mutagenesis protocol.

### Parasite Culture and Transfection.

HFFs were grown in DMEM supplemented with 10% FBS and 2 mM glutamine. *Toxoplasma* tachyzoites were maintained in confluent monolayers of HFF. Epitope-tagged and knockout parasites were generated by transfecting the RH*Δku80Δhxgprt* strain (67), with 15 μg of linearized plasmid and selecting for HXGPRT expression, as previously described (68). The loxP-flanked HXGPRT selection cassette in knockout parasites was removed by transient transfection with a plasmid overexpressing Cre recombinase, and selecting with 6-thioxanthine. WNG1 complement parasites were created by targeting 3xHA-tagged WNG1 (either WT or kinase-dead) driven by its native promoter, together with a bleomycin resistance cassette, to the empty Ku80 locus, and selecting with bleomycin, as previously described (69).

### Immunofluorescence.

HFF cells were grown on coverslips in 24-well plates until confluent and were infected with parasites. The cells were rinsed twice with PBS, and were fixed with 4% paraformaldehyde (PFA)/4% sucrose in PBS at room temperature for 15 min. After two washes with PBS, cells were permeabilized with 0.1% Triton X-100 for 10 min and washed 3× with PBS. After blocking in PBS + 3% BSA for 30 min, cells were incubated in primary antibody in blocking solution overnight at room temperature. Cells were then washed 3× with PBS and incubated with Alexa-Fluor conjugated secondary antibodies (Molecular Probes) for 2 h. Cells were then washed 3× with PBS and then mounted with mounting medium containing DAPI (Vector Laboratories). Cells were imaged on a Nikon A1 Laser Scanning Confocal Microscope. Primary antibodies used in this study include rat anti-HA (1:500 dilution; Sigma), mouse anti-GRA2 (1:1,000 dilution; BioVision), mouse anti-GRA6 (gift of L. D. Sibley, Washington University, St Louis; 1:1,000 dilution), rabbit anti-ROP2 (1:10,000 dilution).

### In Vitro Kinase Assays.

The kinase assays comparing WT and mutant activities were run using 2 µM of His_6_sumo-WNG1, 4 mM MgCl_2_, 200 µM cold ATP, 1 mM DTT, 1 mg/mL BSA, 10% glycerol, 300 mM NaCl, 20 mM Hepes pH 7.5. Reactions were started by adding a hot ATP mix that contained 10 µCi ɣ[^32^P] ATP and 5 µg MBP. The *K*_M,ATP_ kinase assays were run using the same mix as above except nonradioactive ATP was used in a range of concentrations from 1 mM to 32.5 µM. The 25-µL reactions were incubated at a 30 °C water bath for 2 h. Reactions were stopped by adding 9 µL 4× SDS-buffer. Twenty-microliter samples were then run on an SDS/PAGE gel. The gels were Coomassie-stained and the MBP bands were excised and radioactivity quantified using a scintillation counter. All data were analyzed using GraphPad Prism 7.

### Western Blotting.

Proteins were separated by SDS/PAGE and transferred to a PVDF membrane. Membranes were blocked for 1 h in TBST + 5% milk, followed by overnight incubation at 4 °C with primary antibody in blocking solution. The next day, membranes were washed 3× with TBST, followed by incubation at room temperature for 1–2 h with HRP-conjugated secondary antibody (Sigma) in blocking buffer. After three washes with TBST, Western blots were imaged using ECL Plus reagent (Pierce) on a GE ImageQuant LAS4000. Antibodies used in this study include: mouse anti-GRA1 (1:1,000 dilution; BioVision), mouse anti-GRA2 (1:1,000 dilution; BioVision), mouse anti-GRA3 (gift of J.-F. Dubremetz, University of Montpellier, Montpellier, France; 1:2,000 dilution), mouse anti-GRA4 (gift of L. D. Sibley; 1:2,000 dilution), mouse anti-GRA5 (1:1,000 dilution; BioVision), mouse anti-GRA6 (gift of L. D. Sibley; 1:4,000 dilution), rabbit anti-GRA7 (gift of L. D. Sibley; 1:5,000 dilution), rabbit anti-ROP2 (1:10,000 dilution), rat anti-HA (1:1,00 dilution; Sigma). Western blots for quantification were performed independently with each antibody to avoid residual signal due to incomplete stripping.

### Figure Generation.

Structural models were generated using PyMOL v1.7 (70). Secondary structure cartoons were generated using the Pro-origami web server (71). Data plotting and statistical analyses were conducted in Graphpad Prism v7.02. All figures were created in Inkscape v0.91.

## Supplementary Material

Supplementary File

Supplementary File

Supplementary File
