# Supplementary material for: Divergent kinase regulates membrane ultrastructure of the Toxoplasma parasitophorous vacuole
Source: Proc Natl Acad Sci U S A. 2019 Mar 8;116(13):6361–70. doi: 10.1073/pnas.1816161116 (PMC6442604; doi:10.1073/pnas.1816161116)
Supplement: Supplementary File [file pnas.1816161116.sapp.pdf]

PNAS
www.pnas.org

Supplementary Information for

A divergent kinase regulates membrane ultrastructure of the Toxoplasma parasitophorous vacuole

Tsebaot Beraki*, Xiaoyu Hu*, Malgorzata Broncel, Joanna C. Young, William J. O'Shaughnessy, Dominika M. Borek, Moritz Treeck, Michael L. Reese

Address correspondence to Michael L. Reese
Email: michael.reese@utsouthwestern.edu

This PDF file includes:

Supplementary text
Figs. S1 to S7
Tables S1 to S2
References for SI reference citations

Other supplementary materials for this manuscript include the following:

Phosphoproteomics dataset (Dataset S1)
Primers list (Dataset S2)

www.pnas.org/cgi/doi/10.1073/pnas.1816161116

Supplemental Information: Materials & methods

Phylogenetic analysis – Protein sequences for the WNG kinases were identified by using custom scripts that iteratively BLAST (1) the ToxoDBv24 collections for *Toxoplasma gondii*, *Neospora caninum*, *Sarcocystis neurona*, *Eimeria spp.*, *Cystoisospora suis*, and *Cyclospora cayetanensis*. *Besnoitia besnoiti* sequences from the Uniprot nonredundant collection. Multiple sequence alignments were generated by MAFFTv7 (2), and manually edited as necessary. The maximum likelihood phylogenetic tree and bootstrap analysis (1000 replicates) were estimated using RAxML v8.1.17 (3), and the resulting tree was annotated using a script based on the jsPhyloSVG package (4) and Inkscape.

Protein purification – BPK1 (residues 61-377 cloned into pGEX4T) was expressed as a GST fusion in *E. coli* Rosetta2(DE3) overnight at 16°C after induction with 300 mM IPTG. Cells were resuspended in 50 mM Tris 8.0, 200 mM NaCl, 1% Triton-X-100 and 0.2% sodium sarkosyl, lysed by sonication, and centrifuged at 27k rcf for 30 min. GST-fusion protein was affinity purified using glutathione sepharose, which was washed first with PBS containing 1% Triton-X-100, and then without detergent. Protein was eluted by overnight on-bead thrombin cleavage at 4°C overnight. BPK1 was further purified by anion exchange and size exclusion chromatography, where it was flash frozen in 10 mM HEPES, pH 7.0, 100 mM NaCl for storage. Recombinant wild-type and mutant WNG1 (residues 265-591; cloned into pET28) proteins were expressed N-terminally fused with His_6-SUMO in *E. coli* Rosetta2(DE3) incubated overnight at 16°C after induction with 300 mM IPTG. Bacteria were lysed in 50 mM HEPES 7.4, 500 mM NaCl, 15 mM Imidazole, lysed by sonication, and centrifuged as above. His_6-fusion proteins were affinity purified using NiNTA resin, and eluted in 50 mM Tris, pH 7.0, 500 mM NaCl, 250 mM imidazole and dialyzed in 20 mM Tris, pH 7.0, 300 mM NaCl before concentration and flash freezing for long-term storage.

Protein crystallization – Small hexagonal plates of BPK1 grew in a wide variety of conditions in initial screens. High quality crystals were seeded from initial hits grown in 0.2M Proline, 0.1M HEPES

7.4, 10% PEG-3350. To generate a platinum derivative, crystals were soaked with reservoir solution containing 10 mM K_2PtCl_4 for 2 h and washed quickly in reservoir solution. All crystals were flash frozen in a cryoprotectant of reservoir with 25% ethylene glycol.

Data collection, structure determination, and refinement – The diffraction data for the native crystals were collected at beamline 19-ID at the Advanced Photon Source at a wavelength of 1.038 Å and a temperature of 100 K. Native crystals diffracted to 2.5 Å, though diffraction was highly anisotropic, ranging from 2.2 Å in the best dimension to 2.8 Å in the worst. Data for the platinum dervatives were collected in an inverse beam experiment at 1.07195 Å, 1.076276 Å, and 1.07229 Å, corresponding to peak, remote, and inflection wavelengths. Integration, indexing, and scaling of the diffraction data were performed using the HKL2000 suite of programs (5). Initial phases at 3.5 Å were determined by multiwavelength anomalous diffraction from the Pt datasets using the SHELX suite (6) and used to generate a starting model after density modification with the SOLVE/RESOLVE package (7, 8). The high resolution native data were incorporated for extension and map improvement in Phenix (9). Manual rebuilding in Coot (10) and refinement in Refmac5 (11), led to a final 2.5 Å structure of BPK1 (PDB accession: 6M7Z). The structure was evaluated with Molprobity (12).

Homology modeling – A model of the WNG1/ROP35 structure was created in Modeller v9.14 (13) using the BPK1 structure as a template and an alignment of BPK1 and WNG1 created using Clustal Omega (14).

Transmission electron microscopy – Cells were fixed on MatTek dishes with 2.5% (v/v) glutaraldehyde in 0.1M sodium cacodylate buffer. After three rinses in 0.1 M sodium cacodylate buffer, they were post-fixed with 1% osmium tetroxide and 0.8 % $K_3[Fe(CN_6)]$ in 0.1 M sodium cacodylate buffer for 1 h at room temperature. Cells were rinsed with water and en bloc stained with 2% aqueous uranyl acetate overnight. After three rinses with water, specimens were dehydrated with increasing concentration of ethanol, infiltrated with Embed-812 resin and polymerized in a 70ºC oven overnight. Blocks were sectioned with a diamond knife (Diatome) on a Leica Ultracut UC7 ultramicrotome (Leica Microsystems) and collected onto copper grids, post stained with 2% Uranyl acetate in water and lead

citrate. Images were acquired on a Tecnai G2 spirit transmission electron microscope (FEI) equipped with a LaB_6 source at 120 kV. Images were analyzed and quantified using the Fiji distribution of ImageJ (15).

Cell culture, lysis and protein digestion for MS proteomics – All reagents were obtained from Sigma-Aldrich unless specified otherwise. Parental (WT) and RH*Δwng1 Toxoplasma* parasites were cultured in either R0K0 (light) or R10K8 (heavy) SILAC medium (Dundee Cell Products) for 8 generations to ensure efficient heavy label incorporation. 24 h prior cell lysis human foreskin fibroblasts (HFFs) were infected (MOI=5) with WT or RHΔwng1 parasites. Lysis was then performed in 8 M urea, 75 mM NaCl, 50mM Tris, pH=8.2, supplemented with protease (complete mini tablets, Roche) and phosphatase (Phos Stop tablets, Roche) inhibitors followed by sonication to reduce sample viscosity (30 % duty cycle, 3 × 30 sec bursts, on ice). Protein concentration was measured using BCA protein assay kit (Thermo Fisher Scientific) and equal amounts of heavy and light lysates mixed in 1:1 ratio. Lysates were subsequently reduced with 5 mM dithiothreitol (DTT) for 30 min at 56°C and alkylated with 14 mM iodoacetamide for 30 min at room temperature in the dark. Following quenching with 5 mM DTT for 15 min in the dark lysates were diluted with 50 mM ammonium bicarbonate to reduce the concentration of urea to < 2M and digested with trypsin (Promega) overnight at 37 ^{0}C. After digestion samples were acidified with trifluoroacetic acid (TFA) (Thermo Fisher Scientific) to a final concentration of 1 % (v/v), all insoluble material was removed by centrifugation and the supernatant was desalted with Sep-Pak C18 cartridges (Waters). The samples were further digested with LysC (Promega) for 2-3 h at 37 ^{0}C and trypsin overnight at 37 ^{0}C followed by desalting with Sep-Pak as above.

Phosphopeptide enrichment – Desalted and vacuum dried samples were solubilized in 1 ml of loading buffer (80 % acetonitrile, 5 % TFA, 1 M glycolic acid) and mixed with 5 mg of TiO2 beads (Titansphere, 5 µm GL Sciences Japan). Samples were incubated for 10 min with agitation followed by a 1 min 2000 × g spin to pellet the beads. The supernatant containing all non-phosphorylated peptides (total proteome) was removed and stored at -80 ^{0}C. The beads were washed with 150 µl of loading

buffer followed by two additional wash steps, first with 150 µl 80 % acetonitrile, 1 % TFA and second with identical volume of 10 % acetonitrile, 0.2 % TFA. After each wash beads were pelleted by centrifugation (1 min at 2000 × g) and the supernatant discarded. The beads were dried in a vacuum centrifuge for 30 min followed by two elution steps at high pH. For the first elution step the beads were mixed with 100 µl of 1 % ammonium hydroxide (v/v) and for the second elution step with 100 µl of 5 % ammonium hydroxide (v/v). Each time the beads were incubated for 10 min with agitation and pelleted at 2000 × g for 1 min. The two elutions were combined and vacuum dried.

Mass spectrometry sample fractionation and desalting – Both phospho- and total proteome (40 µg) samples were fractionated in a stage tip using Empore SDB-RPS discs (3M). Briefly, each stage tip was packed with one high performance extraction disc, samples were loaded in 100 µL of 1 % TFA, washed with 150 µL of 0.2 % TFA and eluted into 3 fractions with 100 µL of the following: 1) 100 mM ammonium formate, 20 % acetonitrile, 0.5 % formic acid; 2) 200 mM ammonium formate, 40 % acetonitrile, 0.5 % formic acid; 3) 5 % ammonium hydroxide, 60 % acetonitrile. The fractions were taken to dryness by vacuum centrifugation and further desalted on a stage tip using Empore C18 discs (3M). Briefly, each stage tip was packed with one C18 disc, conditioned with 100 µl of 100 % methanol, followed by 200 µl of 1 % TFA. The sample was loaded in 100 µL of 1 % TFA, washed 3 times with 200 µl of 1 % TFA and eluted with 50 µl of 50 % acetonitrile, 5 % TFA. The desalted peptides were vacuum dried in preparation for LC-MS/MS analysis.

nLC-MS/MS and data processing – Samples were resuspended in 0.1 % TFA and loaded on a 50 cm Easy Spray PepMap column (75 µm inner diameter, 2 µm particle size, ThermoFisher Scientific) equipped with an integrated electrospray emitter. Reverse phase chromatography was performed using the RSLC nano U3000 (Thermo Fisher Scientific) with a binary buffer system (solvent A: 0.1 % formic acid, 5 % DMSO; solvent B: 80 % acetonitrile, 0.1 % formic acid, 5 % DMSO) at a flow rate of 250 nl/min. The samples were run on a linear gradient of 2-35 % B in 90 or 155 min with a total run time of 120 or 180 min, respectively, including column conditioning. The nanoLC was coupled to a Q Exactive mass spectrometer using an EasySpray nano source (Thermo Fisher Scientific). The Q

Exactive was operated in data-dependent mode acquiring HCD MS/MS scans (R=17,500) after an MS1 scan (R=70, 000) on the 10 most abundant ions using MS1 target of 1 × 106 ions, and MS2 target of 5 × 104 ions. The maximum ion injection time utilized for MS2 scans was 120 ms, the HCD normalized collision energy was set at 28, the dynamic exclusion was set at 20 or 30 s for 120 and 180 min runs, respectively, and the peptide match and isotope exclusion functions were enabled. Raw data files were processed with MaxQuant (16) (version 1.5.0.25) and peptides were identified from the MS/MS spectra searched against *Toxoplasma gondii* (ToxoDB, 2017) and *Homo sapiens* (Uniprot, 2017) proteomes using Andromeda (17) search engine. SILAC based experiments in MaxQuant were performed using the built-in quantification algorithm (16) with minimal ratio count = 1, enabled 'Match between runs' option for fractionated samples (time window 0.7 min) and 'Re-quantify' feature. Cysteine carbamidomethylation was selected as a fixed modification whereas methionine oxidation, acetylation of protein N-terminus and phosphorylation (S, T, Y) as variable modifications. The enzyme specificity was set to trypsin with maximum of 2 missed cleavages. The precursor mass tolerance was set to 20 ppm for the first search (used for mass re-calibration) and to 4.5 ppm for the main search. The datasets were filtered on posterior error probability to achieve 1% false discovery rate on protein, peptide and site level. "Unique and razor peptides" mode was selected to allow identification and quantification of proteins in groups (razor peptides are uniquely assigned to protein groups and not to individual proteins). Data were further analyzed as described in the Results section and in the Dataset S1 using Microsoft Office Excel 2010 and Perseus v1.5.0.9 (18).

Fractionation of PV membranes – Highly infected monolayers of HFFs were rinsed twice with phosphate buffered saline (PBS) and harvested. PBS containing 1 mM EDTA with protease inhibitors were added to the cells, and cells were mechanically disrupted by passage through a 27 g needle. Proteins secreted in the PV were separated by a low speed (2500 g) spin, and the resulting supernatant (LSS) was further separated by ultracentrifugation at 50,000 rpm for 2 hours at 4°C using a TL100 rotor. The supernatant was aspirated as soluble fraction while the pellet was re-suspended in the same volume buffer. Equal volumes of each fraction were loaded on SDS-PAGE for analysis by

western blot, which were quantified in ImageJ (15).

Triton-X-114 partitioning – The LSS fraction of infected monolayers was prepared as above, and further partitioned using a protocol modified as follows from (19). Pre-condensed Triton-X-114 was added to the LSS to a final concentration of 2% Triton-X-114. After ~5 min incubation on ice, the solution was warmed at 30°C for 3 min, then centrifuged for 5 min at 4000 rpm at room temperature. The top aqueous layer was collected in another tube. The detergent layer was mixed 1:1 with 10 mM Tris-HCl pH7.4, 150 mM NaCl with protease inhibitors and repartitioned, as above. This second aqueous layer was removed and discarded from the detergent enriched fraction. After separation, Triton-X-114 and buffer were added, respectively, to the first aqueous and final detergent phases in order to obtain equal volumes and approximately the same salt and surfactant content for both samples.

Supplemental Figure S1A

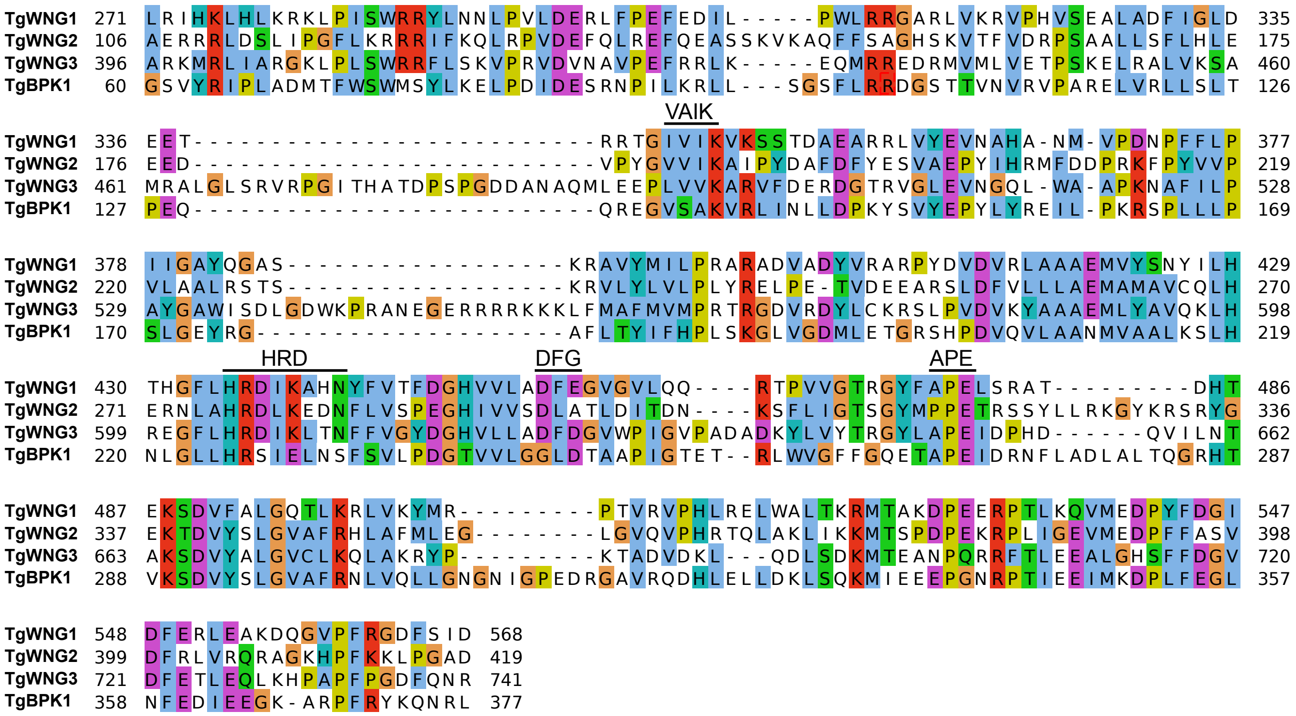

Figure S1A: Alignment of *Toxoplasma gondii* WNG kinase domains. Canonical kinase motifs are indicated above sequences.

Supplemental Figure S1B

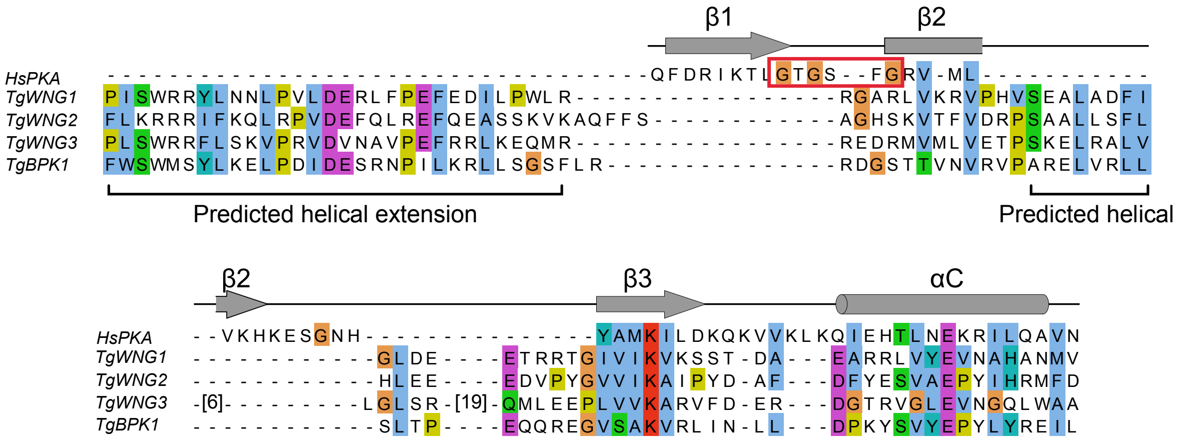

Figure S1B: Alignment of the N-lobes of human PKA and *Toxoplasma gondii* WNG kinase domains. Secondary structure elements from the PKA crystal structure (1ATP) are indicated as cartoons above the alignment. The PKA Gly-loop is boxed in red. Note that the WNG kinases lack sequences corresponding to the Gly-loop, which has been replaced with a conserved sequence predicted to form helices.

Supplemental Table S1C

Name	Organism	Subfamily	Gene Model/Accession	Alternative gene model
CsBPK1	*Cystoisospora suis*	BPK1	CSUI_010109	
HhBPK1	*Hammondia hammondi*	BPK1	HHA_253330	
NcBPK1	*Neospora caninum*	BPK1	NCLIV_007770	
TgBPK1	*Toxoplasma gondii*	BPK1	TGGT1_253330	
BbWNG1	*Besnoitia besnoiti*	WNG1	PFH36021.1	
CsWNG1	*Cystoisospora suis*	WNG1	CSUI_009154	
CsWNG1b	*Cystoisospora suis*	WNG1	CSUI_010099	
EaWNG1	*Eimeria acervulina*	WNG1	EAH_00045380	
EbWNG1	*Eimeria brunetti*	WNG1	EBH_0002260	
EpWNG1	*Eimeria praecox*	WNG1	EPH_0003380	
EtWNG1	*Eimeria tenella*	WNG1	ETH_00005905	
HhWNG1	*Hammondia hammondi*	WNG1	HHA_304740	
NcWNG1	*Neospora caninum*	WNG1	NCLIV_044410	
NcWNG1b	*Neospora caninum*	WNG1	NCLIV_029900	
SnWNG1	*Sarcocystic neurona*	WNG1	SN3_00501335	SRCN_2183
SnWNG1b	*Sarcocystic neurona*	WNG1	SRCN_2123	
TgWNG1	*Toxoplasma gondii*	WNG1	TGGT1_304740	
BbWNG2	*Besnoitia besnoiti*	WNG2	PFH32376.1	
BbWNG2b	*Besnoitia besnoiti*	WNG2	PFH32362.1	
CsWNG2	*Cystoisospora suis*	WNG2	CSUI_004303	
HhWNG2	*Hammondia hammondi*	WNG2	HHA_240090	
NcWNG2	*Neospora caninum*	WNG2	NCLIV_000650	
TgWNG2	*Toxoplasma gondii*	WNG2	TGGT1_240090	
CsWNG2b	*Cystoisospora suis*	WNG2	CSUI_008294	
CsWNG3	*Cystoisospora suis*	WNG3	CSUI_002921	
HhWNG3	*Hammondia hammondi*	WNG3	HHA_201130	
NcWNG3	*Neospora caninum*	WNG3	NCLIV_023260	
SnWNG3	*Sarcocystic neurona*	WNG3	SRCN_4310	SRCN_7082
TgWNG3	*Toxoplasma gondii*	WNG3	TGGT1_201130	
CcWNG4	*Cyclospora cayetanensis*	WNG4	cyc_03158	
EaWNG4	*Eimeria acervulina*	WNG4	EAH_00050320	
EbWNG4	*Eimeria brunetti*	WNG4	EBH_0025260	
EtWNG4	*Eimeria tenella*	WNG4	ETH_00026495	
BbWNG5	*Besnoitia besnoiti*	WNG5	PFH31612.1	

Table S1C: Gene models (for sequences in ToxoDB) or NCBI accession numbers of sequences used in this study.

Figure S2

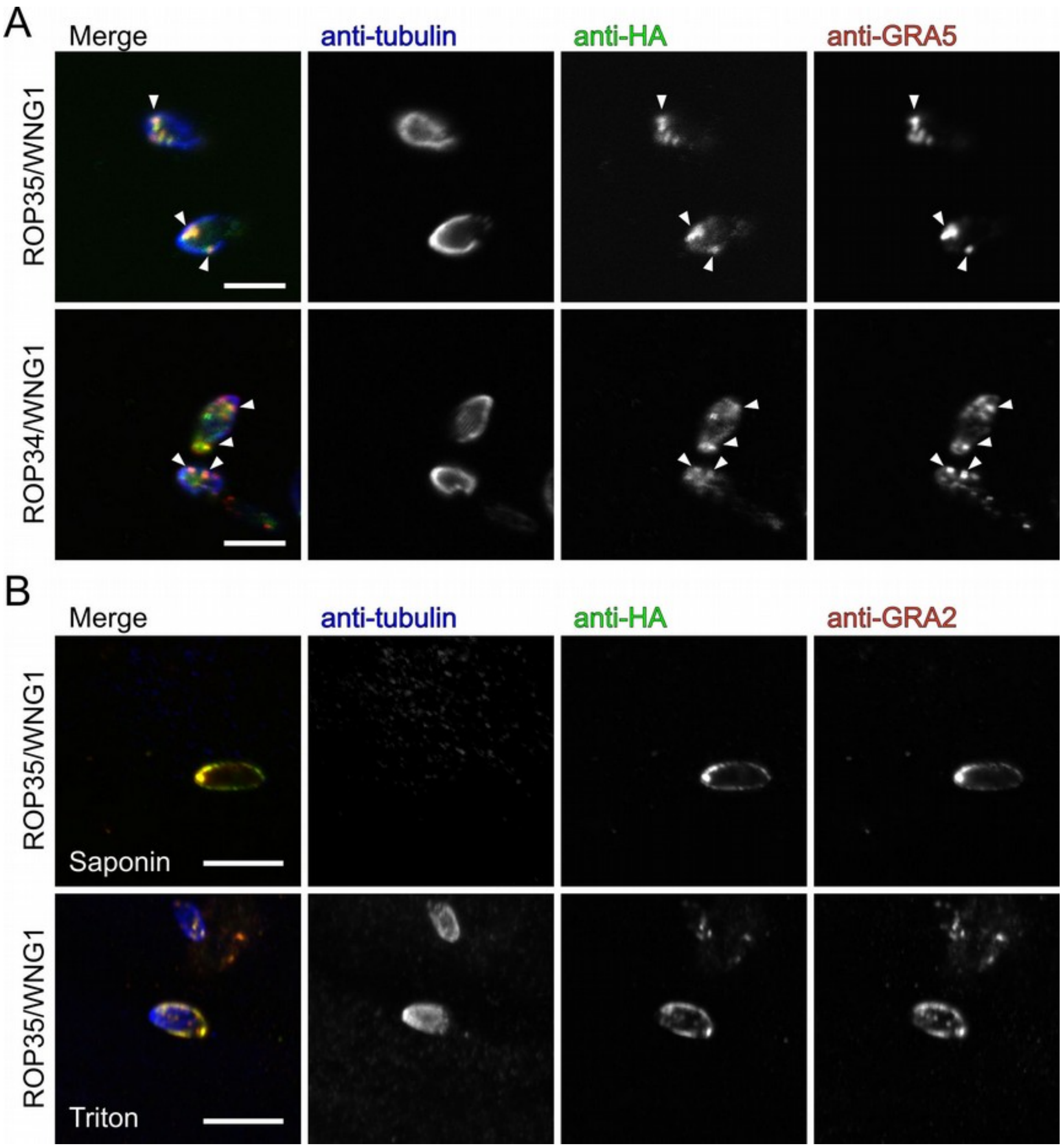

Figure S2: WNG kinases localize to the dense granules. (A) 0.5 μm confocal slices of ROP35/WNG1-3xHA or ROP34/WNG2-3xHA extracellular parasites stained with anti-β-tubulin (blue), anti-HA (green), and the dense granule marker GRA5 (red). Arrowheads indicate punctate co-localization of the HA and GRA5 signal. (B) ROP35/WNG1-3xHA (green) co-localizes with GRA2 (red) in a newly formed parasitophorous vacuoles within 10 minutes after infection. Parasites were permeabilized with either 0.001% saponin (which did not permeabilize the parasite plasma membrane; note lack of anti-tubulin signal) or 0.1% Triton-X-100. Scale bars: 10 μm.

Supplemental Figure S3

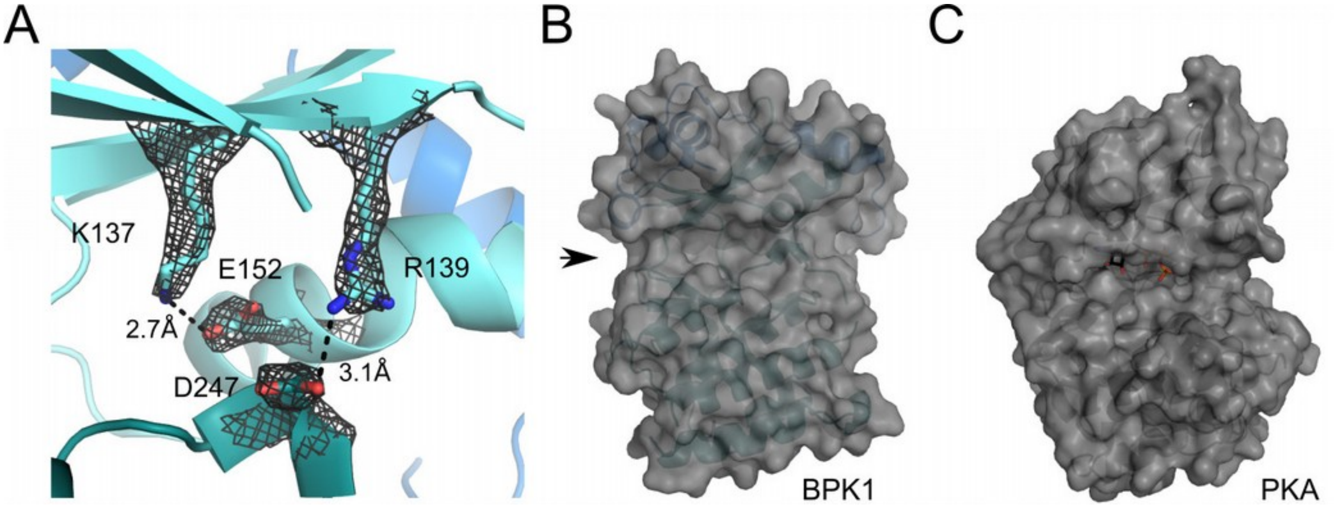

Figure S3: BPK1 has a divergent, open active site. (A) BPK1 active site superposed with the $2F_O$–F_C electron density map contoured at 2σ. Two salt bridges are highlighted as sticks: the conserved bridge between the αC E152 and the VAIK K137 as well as an unusual, WNG family-specific salt bridge between R139 and D247 (an acidic substitution at the DFG Gly position). The lack of Gly-loop creates an open active site in BPK1, indicated with an arrow in (B). This is compared to the more restricted active site in canonical kinases such as PKA, shown in (C). Note that the two kinases are shown in equivalent orientations.

Supplemental Table S2: Crystallographic Data and Refinement

	TgBPK1 (native)	TgBPK1 (Pt)		
**Data collection**				
Space group	$P2_12_12$	$P2_12_12$		
Cell dimensions				
a, *b*, *c* (Å)	171.47, 123.07, 86.62	184.77, 120.98, 92.36		
α, β, γ (°)	90, 90, 90	90, 90, 90		
		Peak	Inflection	Remote
Wavelength (Å)	1.038	1.07195	1.07229	1.076276
Resolution (Å)	47.7 – 2.50 (2.59 – 2.50)	50.0 – 3.75 (3.81 – 3.75)	50.0 – 3.73 (3.79 – 3.73)	50.0 – 3.92 (3.92 – 3.92)
Total reflections	512518	130864	110497	105514
R_{merge}	9.1 (86.3)	8.3 (1.0)	7.4 (88.2)	8.6 (96.6)
$CC_{1/2}$ (final shell)	0.76	0.63	0.70	0.75
$I / I\sigma$	23.8 (2.0)	25.2 (1.8)	25.3 (2.0)	24.4 (2.2)
†Completeness (%)	90.9 (63.5)	90.3 (61.1)	87.2 (63)	89.8 (60.2)
Redundancy	8.0 (7.3)	6.0 (6.1)	5.0 (5.1)	6.0 (5.9)
**Refinement**				
No. reflections	58472 (4024)			
R_{free} reflections	2987 (203)			
R_{work} / R_{free}	0.197 / 0.238 (0.238 / 0.289)			
No. atoms				
Protein	12422			
Ligand/ion	64 (EDO), 3 (CL)			
Water	381			
B-factors				
Protein	45.58			
Ligand/ion	47.55			
Water	33.12			
R.m.s. deviations				
Bond lengths (Å)	0.010			
Bond angles (°)	1.37			
Ramachandran (favored/disallowed)	98.48 / 0			
Molprobity score	1.14			
Molprobity clash score	3.48			
No. TLS groups	20 per chain			

†Diffraction was anisotropic, which reduced the completeness, especially in highest resolution shell; for instance, for the native dataset diffraction was measured to 2.2 Å in the strongest dimension and 2.8 Å in the weakest.

Supplemental Figure S4

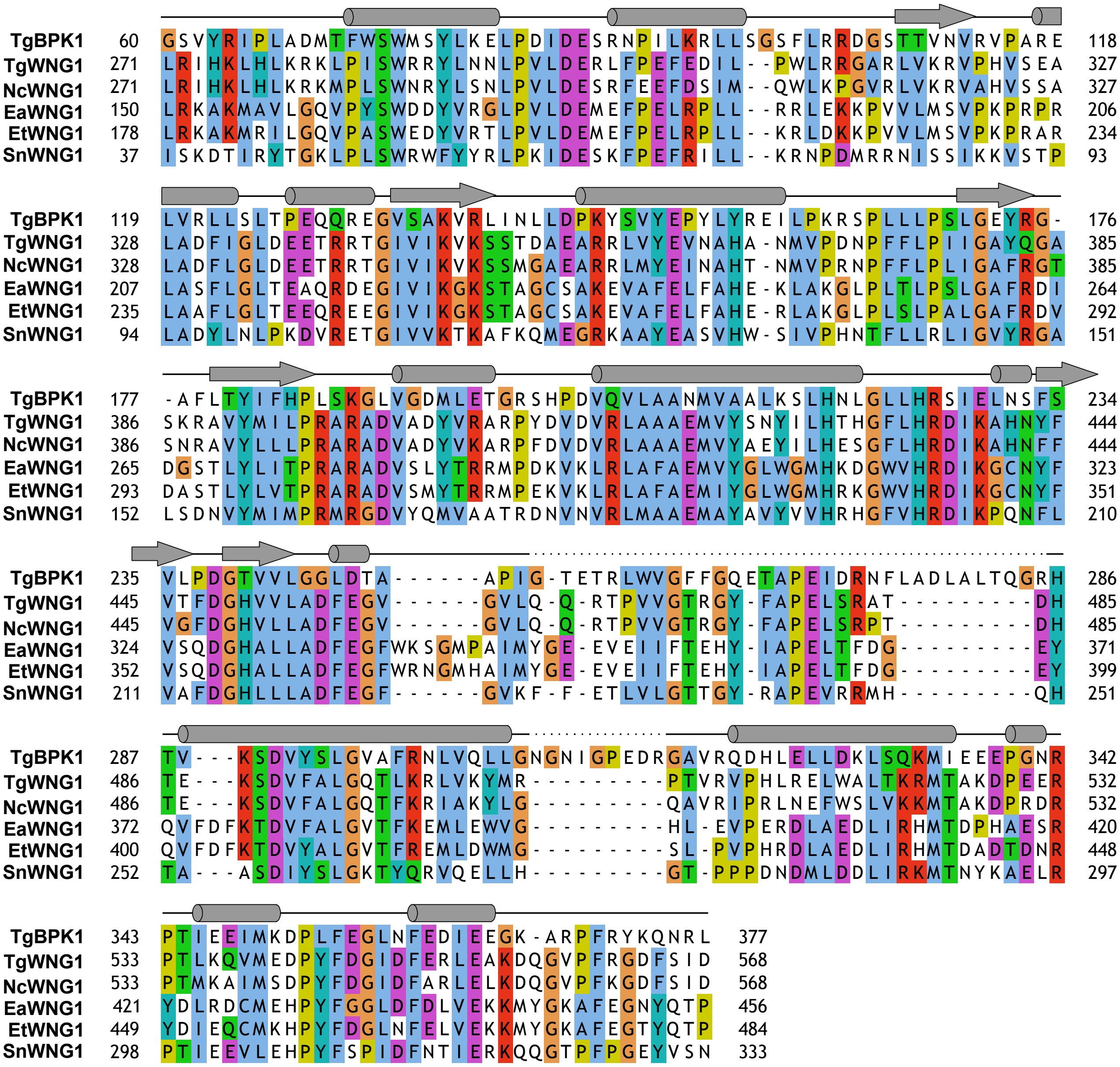

Figure S4: Multiple sequence alignment of WNG1 kinase domains with TgBPK1. The secondary structure from the TgBPK1 crystal structure are shown in cartoon above the alignment. Dashed lines indicate a lack of density corresponding to the indicated sequence.

Supplemental Figure S5a

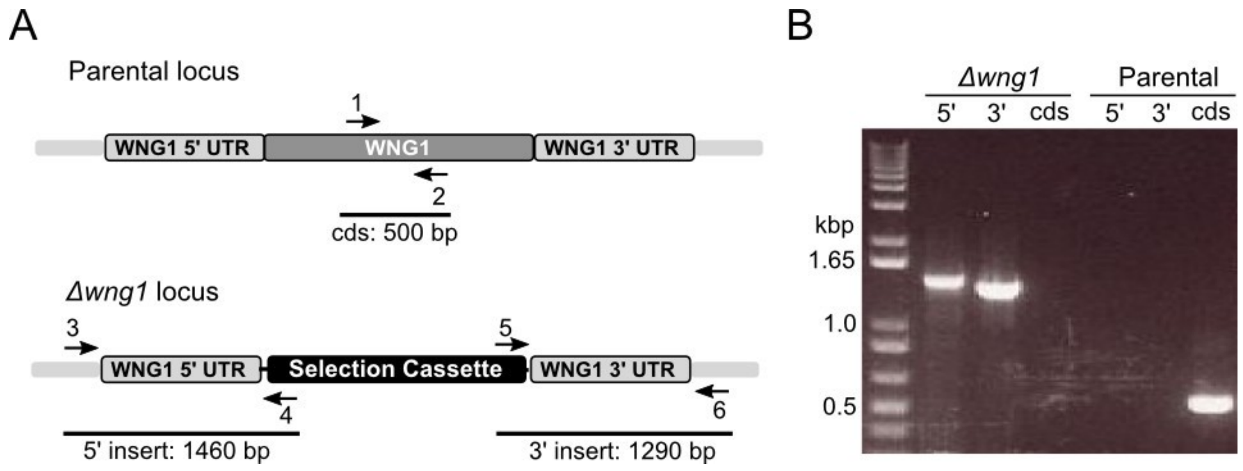

Figure S5a: Generation of WNG1 knockout parasites. RH*Δwng1* parasites were generated by double homologous recombination in which the WNG1 genomic sequence was replaced by a HXGPRT selection cassette. (A) Cartoon of parental and knockout loci indicating binding sites for primers used to verify knockout. (B) PCR demonstrating insertion of selection cassette and loss of coding sequence (cds) in knockout parasites. Primers sequences are listed in Dataset S2.

Supplemental Figure S5b

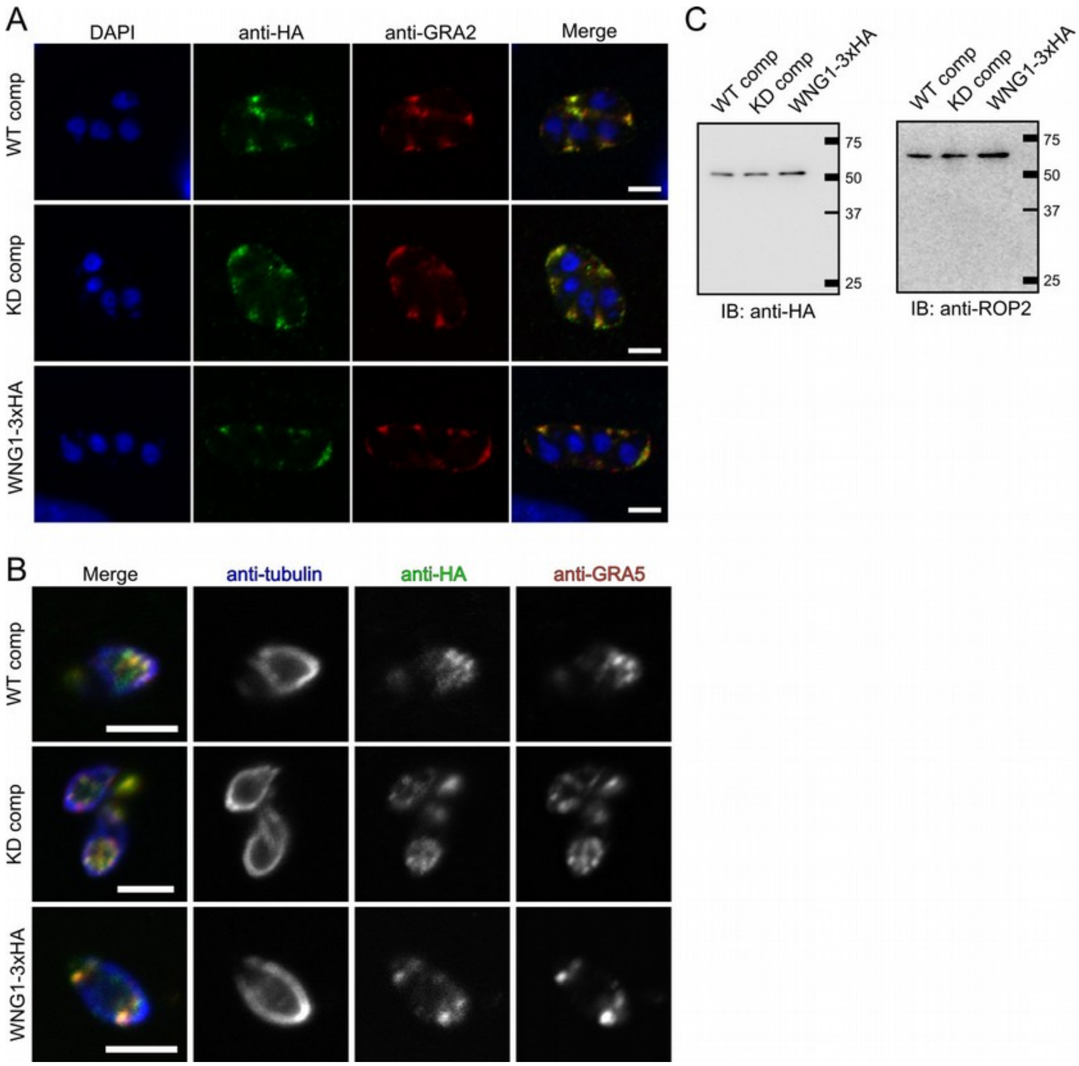

Figure S5b: WNG1 complements faithfully localize to dense granules and PV. (A) 0.5 μm confocal slices of the wild-type (WT) and kinase-dead (KD) WNG1 complemented parasites as well as the endogenously tagged WNG1-3xHA were stained with DAPI (blue), anti-HA (green), and the dense granule and IVN marker GRA2 (red). (B) 0.5 μm confocal slices of the indicated extracellular parasites stained with anti-tubulin (blue), anti-HA (green), and anti-GRA5 (red). (C) Both the WT and KD WNG1-complements are expressed at similar levels to the endogenously 3xHA tagged protein, as demonstrated by western blot, using ROP2 as a loading control.

Supplemental Figure S5c

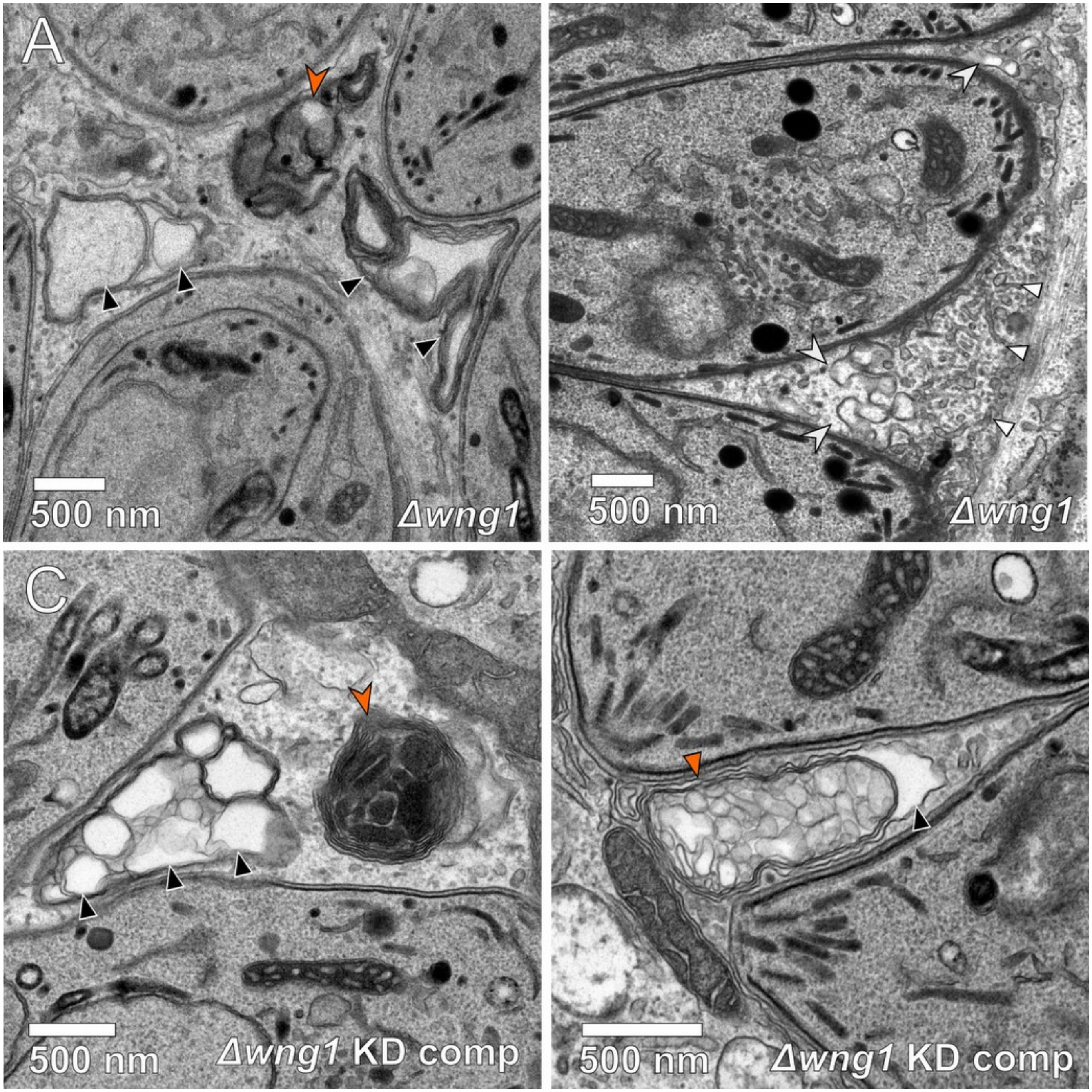

Figure S5c: Unusual membrane structures in vacuoles lacking active WNG1 kinase. Representative transmission electron microscopic images of (A,B) RH*Δwng1* and (C,D) RH*Δwng1* complemented with kinase-dead WNG1. IVN tubules are indicated with small white triangles. Multilamellar vesicles are indicated with small solid orange triangles. Multilamellar structures in which internal vesicles appear to have been lost during fixation or to have collapsed into sheets are indicated with black triangles. Electron dense multilamellar structures are indicated with a large orange arrowheads in (A) and (C). Membrane "whirls" that appear connected with IVN tubules are indicated with large white arrowheads in (B).

Supplemental Figure S6

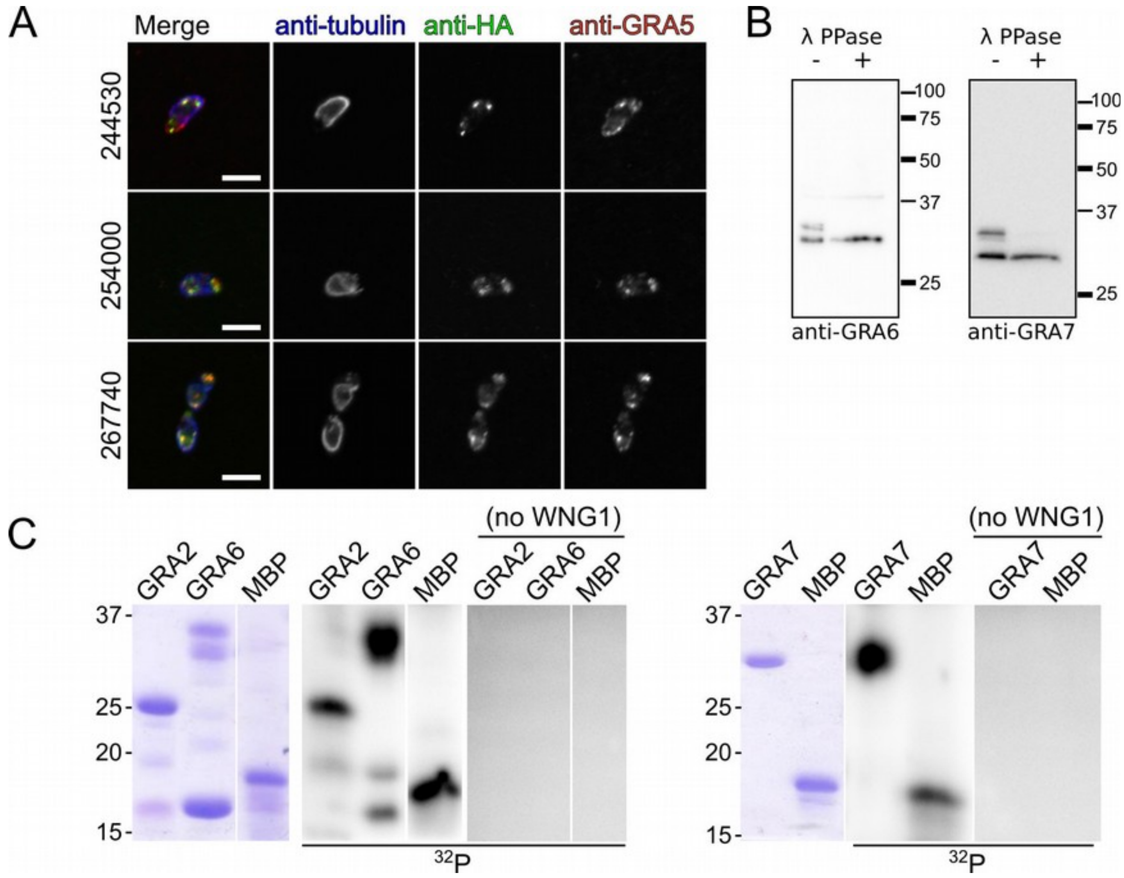

Figure S6: WNG1 substrates are dense granule proteins. (A) 0.5 μm confocal slices of extracellular parasites with the indicated candidate WNG1 substrate endogenously tagged with 2xHA and stained with anti-HA (green), anti β-tubulin (blue), and anti-GRA5 (red). (B) The slower migrating GRA6 and GRA7 bands correspond to phosphorylated species. Lysate from cells infected with parasites with a wild-type WNG1 locus were either treated with λ-phosphatase (+) for 30 minutes at 30°C or left untreated (-) and western blotted with the indicated antibody. (C) Bacterially expressed His_6-tagged GRA2, GRA6, and GRA7 were purified on NiNTA resin and used as substrates for a WNG1 kinase assay with $\gamma[^{32}P]$-ATP and visualized by autoradiogram. 4 μg MBP was used as a positive control. Coommassie stained gels indicate relative protein amounts used in the assay.

Supplemental Figure S7

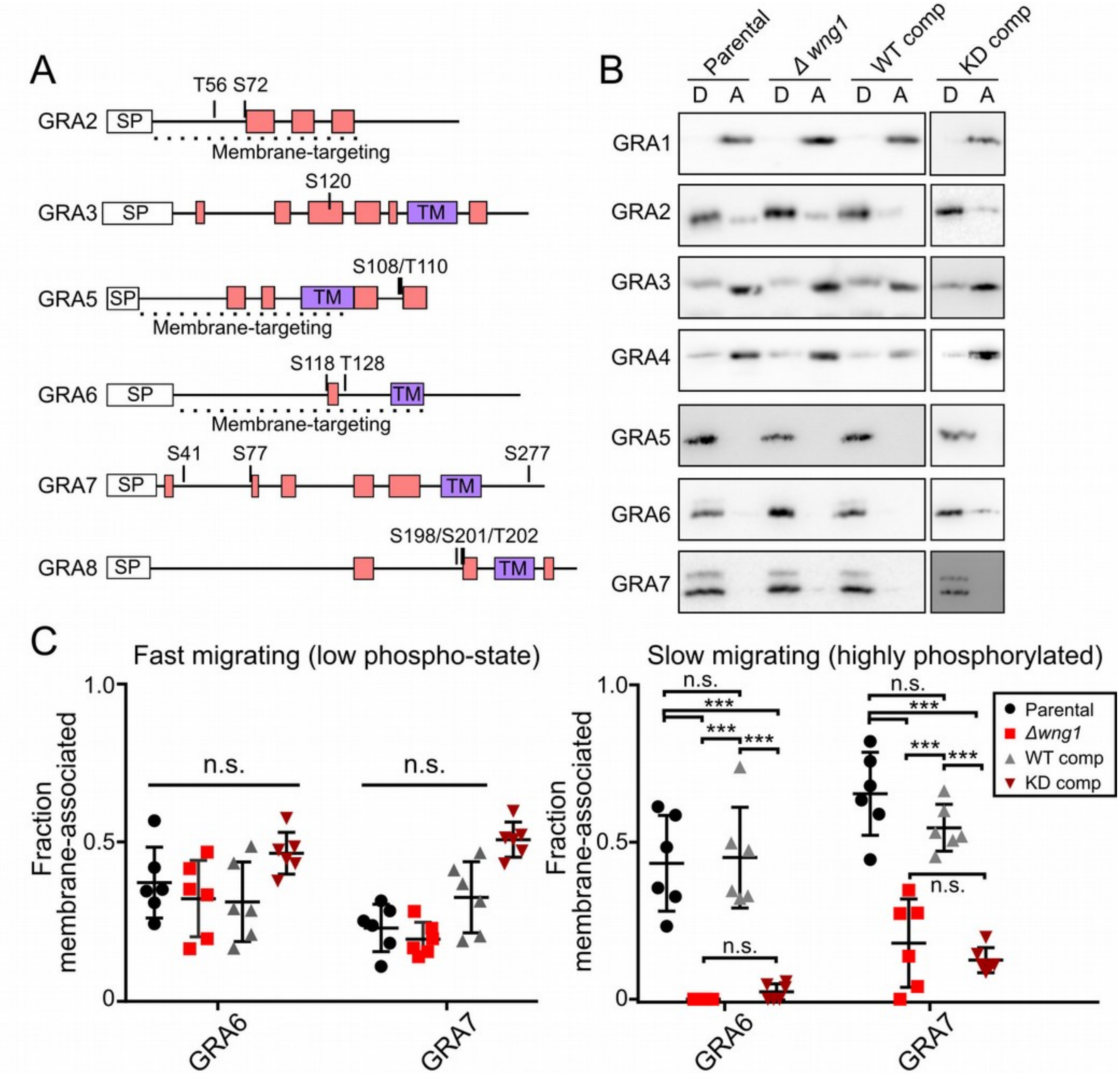

Figure S7: WNG1 activity does not affect TX-114 partitioning of IVN GRA proteins. (A) WNG1-dependent phosphosites are mapped onto the predicted secondary structures of the indicated GRA proteins. Predicted α-helices are shown as rectangles. Predicted transmembrane helices (TM) are shaded purple. (B) Host and PV membranes from cells infected with the indicated strains were partitioned in TX-114 and the detergent [D] and aqueous [A] phases were separated by SDS-PAGE and analyzed by western blot probed with antibodies to the indicated GRA proteins. We note that

GRA2 and GRA3 appear to exhibit a slightly slower electrophoretic mobility in the detergent-phase samples. This may reflect differences in the post-translationally modified states of the proteins in the different samples. While detergent concentrations were normalized between samples, this also may be due to an artifact of the proteins' behavior in the SDS-PAGE. (C) Data from Figure 8 were requantified to compare the fractional membrane-association of the faster migrating, unphosphorylated species (left panel) with the slower migrating, highly phosphorylated species (right panel) of GRA6 and GRA7 in vacuoles of the parental, knockout, and complement strains. Significance was analyzed by ANOVA with Tukey's test (***, $p < 0.001$; n.s., not significant). Note that while the difference between the fast migrating GRA7 band from kinase-dead WNG1 complemented vacuoles appears to be borderline significant ($p \sim 0.05$) when compared to the other samples; it is unlikely to be biologically relevant.

Supplemental References

1. Camacho C, et al. (2009) BLAST+: architecture and applications. *BMC Bioinformatics* 10:421.
2. Katoh K, Standley DM (2013) MAFFT multiple sequence alignment software version 7: improvements in performance and usability. *Mol Biol Evol* 30(4):772–780.
3. Stamatakis A (2014) RAxML version 8: a tool for phylogenetic analysis and post-analysis of large phylogenies. *Bioinforma Oxf Engl* 30(9):1312–1313.
4. Smits SA, Ouverney CC (2010) jsPhyloSVG: a javascript library for visualizing interactive and vector-based phylogenetic trees on the web. *PloS One* 5(8):e12267.
5. Otwinowski Z, Minor W (1997) Processing of X-ray diffraction data collected in oscillation mode. *Methods Enzymol* 276:307–326.
6. Sheldrick GM (2008) A short history of SHELX. *Acta Crystallogr A* 64(Pt 1):112–122.
7. Terwilliger TC, Berendzen J (1999) Automated MAD and MIR structure solution. *Acta Crystallogr D Biol Crystallogr* 55(Pt 4):849–861.
8. Terwilliger T (2004) SOLVE and RESOLVE: automated structure solution, density modification and model building. *J Synchrotron Radiat* 11(Pt 1):49–52.
9. Adams PD, et al. (2010) PHENIX: a comprehensive Python-based system for macromolecular structure solution. *Acta Crystallogr D Biol Crystallogr* 66(Pt 2):213–221.
10. Emsley P, Lohkamp B, Scott WG, Cowtan K (2010) Features and development of Coot. *Acta Crystallogr D Biol Crystallogr* 66(Pt 4):486–501.

11. Winn MD, Murshudov GN, Papiz MZ (2003) Macromolecular TLS refinement in REFMAC at moderate resolutions. *Methods Enzymol* 374:300–321.

12. Chen VB, et al. (2009) *MolProbity* : all-atom structure validation for macromolecular crystallography. *Acta Crystallogr D Biol Crystallogr* 66(1):12–21.

13. Sali A, Blundell TL (1993) Comparative protein modelling by satisfaction of spatial restraints. *J Mol Biol* 234(3):779–815.

14. Sievers F, et al. (2011) Fast, scalable generation of high-quality protein multiple sequence alignments using Clustal Omega. *Mol Syst Biol* 7:539.

15. Schindelin J, et al. (2012) Fiji: an open-source platform for biological-image analysis. *Nat Methods* 9(7):676–682.

16. Cox J, Mann M (2008) MaxQuant enables high peptide identification rates, individualized p.p.b.-range mass accuracies and proteome-wide protein quantification. *Nat Biotechnol* 26(12):1367–1372.

17. Cox J, et al. (2011) Andromeda: a peptide search engine integrated into the MaxQuant environment. *J Proteome Res* 10(4):1794–1805.

18. Tyanova S, et al. (2016) The Perseus computational platform for comprehensive analysis of (prote)omics data. *Nat Methods* 13(9):731–740.

19. Bordier C (1981) Phase separation of integral membrane proteins in Triton X-114 solution. *J Biol Chem* 256(4):1604–1607.
